# Ultrafast three-dimensional microbubble imaging *in vivo* predicts tissue damage volume distributions during nonthermal brain ablation

**DOI:** 10.7150/thno.47281

**Published:** 2020-06-01

**Authors:** Ryan M. Jones, Dallan McMahon, Kullervo Hynynen

**Affiliations:** 1 Physical Sciences Platform, Sunnybrook Research Institute, Toronto, Ontario, Canada.; 2 Department of Medical Biophysics, University of Toronto, Toronto, Ontario, Canada.; 3 Institute of Biomaterials and Biomedical Engineering, University of Toronto, Toronto, Ontario, Canada.

**Keywords:** image-guided therapy, focused ultrasound, microbubble contrast agents, nonthermal ablation, ultrafast 3D acoustic imaging

## Abstract

Transcranial magnetic resonance imaging (MRI)-guided focused ultrasound (FUS) thermal ablation is under clinical investigation for non-invasive neurosurgery, though its use is restricted to central brain targets due primarily to skull heating effects. The combination of FUS and contrast agent microbubbles greatly reduces the ultrasound exposure levels needed to ablate brain tissue and may help facilitate the use of transcranial FUS ablation throughout the brain. However, sources of variability exist during microbubble-mediated FUS procedures that necessitate the continued development of systems and methods for online treatment monitoring and control, to ensure that excessive and/or off-target bioeffects are not induced from the exposures.

**Methods:** Megahertz-rate three-dimensional (3D) microbubble imaging *in vivo* was performed during nonthermal ablation in rabbit brain using a clinical-scale prototype transmit/receive hemispherical phased array system.

**Results:** *In-vivo* volumetric acoustic imaging over microsecond timescales uncovered spatiotemporal microbubble dynamics hidden by conventional whole-burst temporal averaging. Sonication-aggregate ultrafast 3D source field intensity data were predictive of microbubble-mediated tissue damage volume distributions measured post-treatment using MRI and confirmed via histopathology. Temporal under-sampling of acoustic emissions, which is common practice in the field, was found to impede performance and highlighted the importance of capturing adequate data for treatment monitoring and control purposes.

**Conclusion:** The predictive capability of ultrafast 3D microbubble imaging, reported here for the first time, will enable future microbubble-mediated FUS treatments with unparalleled precision and accuracy, and will accelerate the clinical translation of nonthermal tissue ablation procedures both in the brain and throughout the body.

## Introduction

Focused ultrasound (FUS) offers a non-invasive approach for depositing localized acoustic energy deep into the body, which can be harnessed to elicit a wide range of biological effects on vasculature and tissue for therapeutic purposes 1. Given the potential risks associated with invasive interventions in the brain, there has been a longstanding interest in the use of FUS for non-invasive brain therapy (*e.g.*, thermal ablation 2,3), yet for many years the technique's clinical translation was considered infeasible due to complications related to the intervening skull 4. Nevertheless, the development of large-scale phased array transducers 5, modeling-based transcranial aberration correction strategies 6, and magnetic resonance imaging (MRI) methods to spatially map quantitative temperature changes within the body 7 have enabled controlled FUS delivery through the intact skull for thermal therapy, thereby renewing interest in this technology for neurological indications. Transcranial MRI-guided FUS thermal ablation has since been shown to be clinically feasible within the midbrain, where maximal acoustic intensities can be delivered through the skull with minimal cranial heating 8,9, but not at less central brain regions. This emerging surgical technique has now been approved by the U.S. Food and Drug Administration for both medication-refractory essential tremor 10 and tremor-dominant Parkinson's disease 11, and is under clinical investigation for the treatment of brain tumors 12, chronic pain 13, and psychiatric conditions 14, indications for which the associated targets are all located centrally in the brain. However, peripheral tumors and disorders that require off-central (*e.g.*, epilepsy 15) or near skull-base (*e.g.*, trigeminal neuralgia 16) targets remain impossible to treat via FUS thermoablation.

In addition to thermal therapies, FUS exposures can be combined with intravenously administered microbubble contrast agents that can oscillate and collapse in response to ultrasound stimulation to produce a variety of mechanical bioeffects that are concentrated within the vasculature 1. One promising application of microbubble-mediated ultrasonic therapy is for inducing transient and localized blood-brain barrier (BBB) permeability enhancement 17 to enable targeted delivery of therapeutics into the central nervous system (CNS), a technique that recently entered clinical testing in patients with brain tumors 18-20, Alzheimer's disease 21, and amyotrophic lateral sclerosis 22. With stronger exposure conditions that are still well below the threshold for generating thermal coagulation, the induced microbubble behavior (*i.e.*, acoustic cavitation 23) can be violent enough to damage and destroy capillaries, ultimately leading to infarct and tissue necrosis 24. Because the time-averaged power levels required for such 'nonthermal' ablation are approximately two orders of magnitude below those needed for thermal ablation, this approach has been of particular interest for expanding the 'treatment envelope' of transcranial FUS brain surgery 25-29. Microbubble-mediated nonthermal ablation has been investigated in brain 24-29 and tumour 30-35 tissue in pre-clinical models but has yet to be tested in humans.

Widespread clinical adoption of cavitation-mediated FUS therapies will require the continued development of systems and methods for online treatment monitoring and control, to ensure that safe yet effective acoustic exposure levels are maintained throughout the sonications 36. The biological outcomes resulting from ultrasound-stimulated microbubble activity *in vivo* are dependent on several factors related to the sonication scheme (*i.e.*, frequency, peak negative pressure, burst length, burst repetition frequency, exposure duration), the microbubble contrast agent (*e.g.*, gas core and shell composition, size distribution, administration method), and the local environment (*e.g.*, tissue/tumor vascularity, blood-oxygen level, blood viscosity, ambient over-pressure) 37. Moreover, the range of exposure conditions over which the desired bioeffects can be induced within the targeted region(s) without causing excessive and/or off-target cavitation effects is relatively narrow 28,29,38-41, with potential for blood vessel rupture in cases of over-exposure 42. Furthermore, FUS parameter selection is challenging during brain applications as substantial variability in transcranial ultrasound transmission efficiency 43,44 and imperfect trans-skull focusing via current aberration correction approaches 6 complicate estimation of the pressure fields generated *in situ* from a fixed transducer output. Taken together, these sources of treatment variability necessitate continuous, real-time monitoring of FUS-induced microbubble activity throughout the exposures to ensure the safety and efficacy of the procedures.

Remote detection of the acoustic emissions radiated by microbubbles in response to ultrasound stimulation provides valuable feedback during bubble-mediated FUS procedures, as analysis of these signals can be used to characterize aspects of the underlying bubble oscillation dynamics 23. In pre-clinical studies the acoustic signals detected during FUS exposures with circulating microbubbles have been identified as potential indicators of biological outcome to therapy 38,39,45 and have subsequently been investigated for online treatment monitoring and control 41,46-48. The current commercial transcranial FUS brain system (ExAblate Neuro, InSightec Inc., Tirat Carmel, Israel) is equipped with dedicated single-element cavitation detectors for real-time treatment feedback; during the initial clinical trials of trans-skull FUS-induced BBB permeability enhancement 19-22 the captured microbubble emissions are being employed for exposure level calibration 46 and sonications are terminated automatically by the system's controlling software if signatures of excessive bubble activity are observed 49. However, because single-element detectors provide limited information regarding the spatial distribution of ultrasound-stimulated microbubble activity, the acquired acoustic emissions must be assumed to have originated from the prescribed target region(s). Consequently, this approach does not allow monitoring of potential unwanted bioeffects generated outside of the intended treatment region(s), which have been observed in animal studies following FUS exposures with microbubbles in circulation 25,28,41.

The use of multi-element detector arrays combined with passive cavitation imaging (PCI) techniques can provide spatial information regarding microbubble activity in the brain 47,50-57 that can be exploited for FUS exposure level calibration 47, and element-specific aberration corrections can be incorporated into the beamforming process to augment image quality in transcranial scenarios 51,52,58-60. Previous pre-clinical studies that have attempted to correlate characteristics of *in-vivo* PCI data with FUS-induced bioeffects in the brain have employed narrow-aperture one-dimensional (1D) receiver arrays for acoustic signal acquisition 50,55-57. Narrow-aperture 1D arrays suffer from poor spatial resolution during passive beamforming and are limited to imaging two-dimensional (2D) regions located near the central axis of the device 61,62, which has restricted prior analyses to small field-of-view (FOV) 2D, 1D, or binary correlations 50,55-57. Moreover, in these studies acoustic emissions data were acquired over a small fraction of the total FUS on-time (*i.e.*, ~0.01-6.0% at the start of each 6.7-10 ms burst 50,55-57), which may have further limited the predictive capability of the resulting 2D PCI data.

Large-aperture 2D receiver arrays enable three-dimensional (3D) imaging with improvements in spatial resolution, receive sensitivity, and the effective imaging volume during passive beamforming 47,51-54,58, which is expected to improve predictions of the bioeffect distributions induced during microbubble-mediated FUS therapies. In the current study, the feasibility of using 3D PCI data to predict the volumetric tissue damage distributions induced during microbubble-mediated nonthermal brain ablation is demonstrated for the first time. Using a clinical-scale multi-frequency sparse hemispherical transmit/receive phased array 47, burst-mode FUS was applied to rabbit brain at different pressure amplitudes and acoustic emissions data were acquired simultaneously over the total duration of FUS on-time. Retrospective short-time analysis of the acquired acoustic emissions data was performed to investigate the intra-burst spatiotemporal evolution of microbubble activity *in vivo* over microsecond timescales*,* and was compared with conventional whole-burst temporal-average processing. Different methods of combining ultrafast 3D microbubble imaging data acquired throughout the exposures were contrasted by evaluating the predictive capability of sonication-aggregate 3D source field intensity data for detecting the tissue damage volume distributions measured post-treatment using MRI and confirmed via histopathology. The impact of the acoustic emissions acquisition window (*i.e.*, variable onset and duration) on performance was examined retrospectively to provide signal acquisition strategies for scenarios in which full-sampling is not feasible (*e.g.*, due to hardware limitations). The acoustic feedback provided by ultrafast 3D microbubble imaging will enable full volumetric control of treatment response during microbubble-mediated FUS therapies, and will accelerate the clinical translation of nonthermal tissue ablation procedures both in the brain and throughout the body.

## Results

### Multi-point 3D subharmonic imaging calibration is feasible *in vivo*

**Figure 1:**
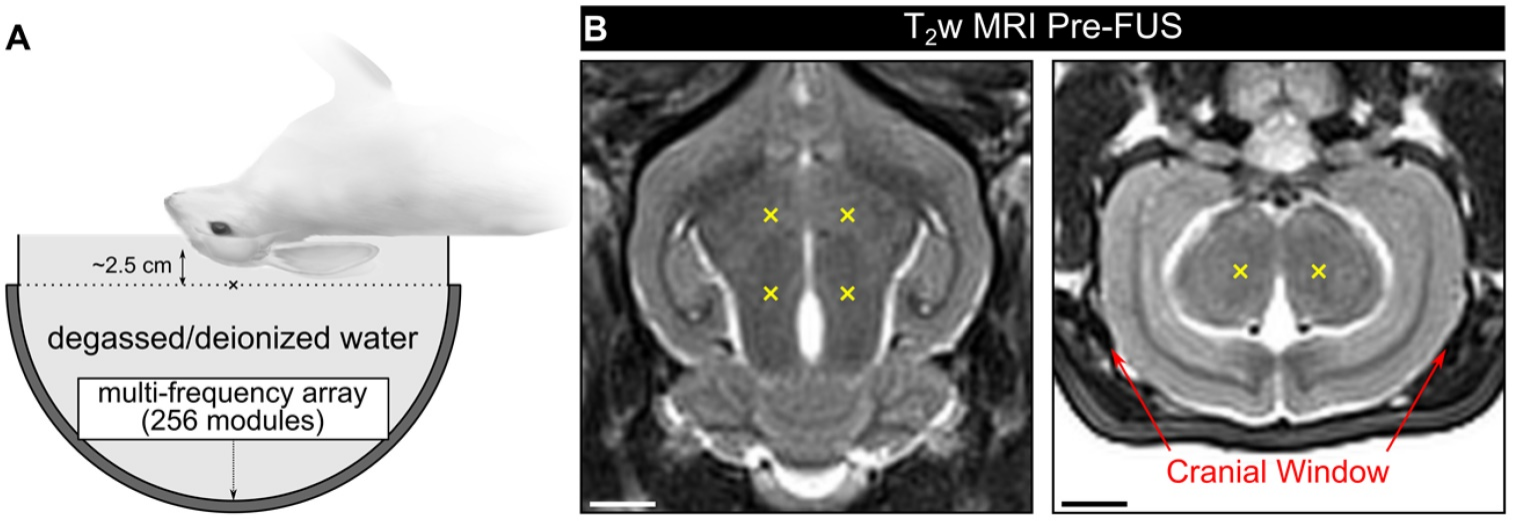
** Experimental setup.** (**A**) Rabbits were laid supine on a supporting platform with their heads immobilized, coupled acoustically to the FUS system via degassed/deionized water, and positioned such that an axial target plane within the mid-thalamus was centered on the hemispherical transducer's acoustic axis ~2.5 cm distal to the array's geometric focus ('x' symbol). (**B**) Pre-sonication T_2_w MRI data (axial and coronal planes, rabbit #5) with target locations for a 2 x 2 point grid (6 mm point spacing) overlaid in yellow ('x' symbols). When viewing the axial plane, the beam steering was performed in a clockwise pattern and the sonication direction was into the page. Red arrows on the coronal plane indicate the extent of the cranial windows (cohort #1). Scale bars indicate 5 mm.

Experiments were conducted in a rabbit model using a multi-frequency transmit/receive ultrasound phased array system (**Figure 1A**). In the first cohort of animals (cohort #1), burst-mode FUS (***f***_0_ = 612 kHz, burst length = 10 ms, burst repetition frequency = 1 Hz) was electronically steered over four targets within the mid-thalamus of craniotomized rabbits (**Figure 1B**), and a 3D subharmonic imaging-based feedback approach 47 modified to enable multi-point exposure level calibration was employed. No subharmonic activity was detected during any of the multi-point baseline ramp sonications performed prior to microbubble administration (maximum spatial-peak temporal-peak negative (SPTPN) pressure ~1.2 MPa *in situ*), consistent with our previous work with a similar experimental setup 47.

**Figure 2:**
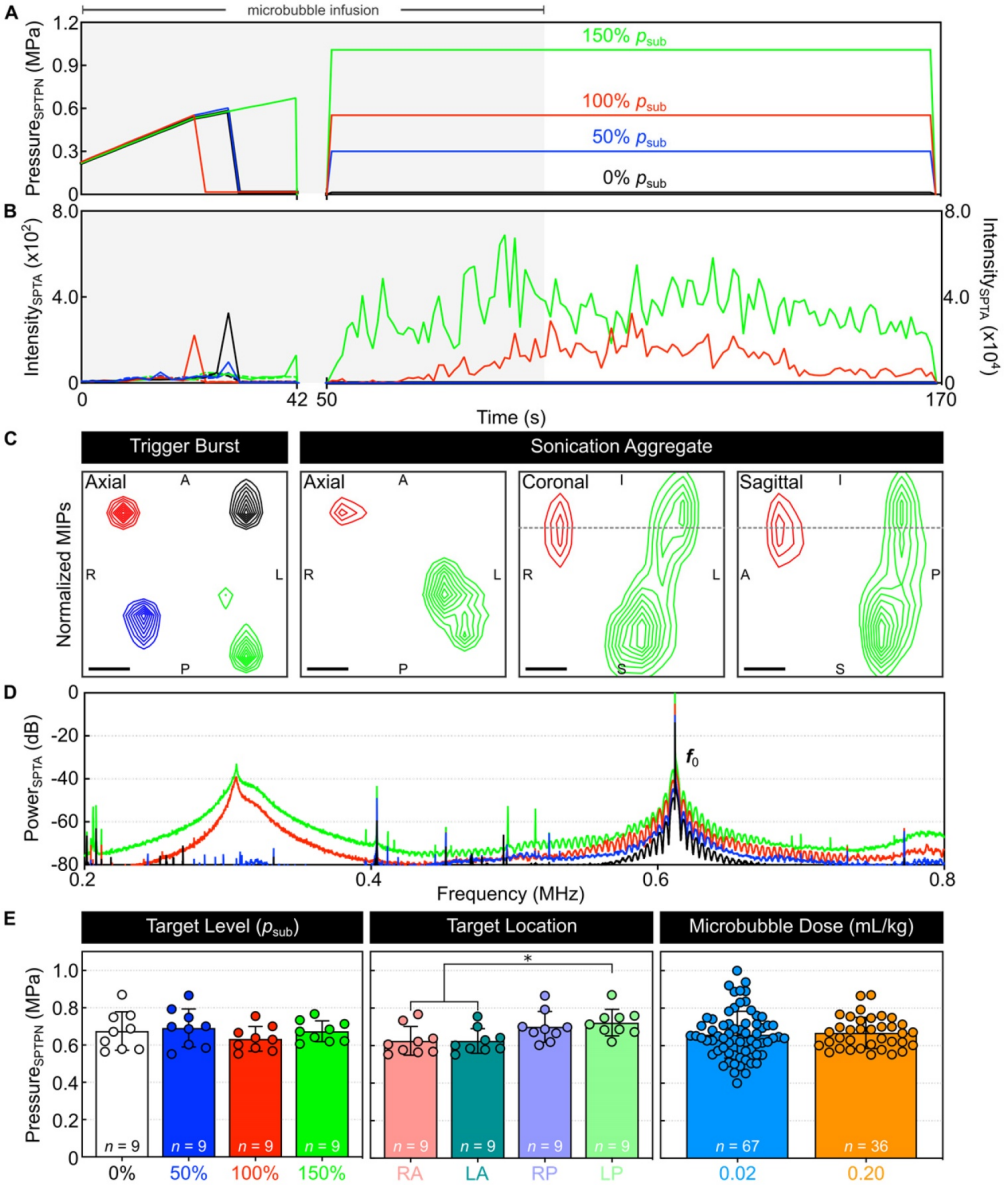
** Multi-point 3D subharmonic imaging calibration *in vivo*.** (**A**) Estimated *in-situ* SPTPN pressure and (**B**) SPTA source field intensity as a function of time during a four-point sonication (0%, 50%, 100%, and 150% *p*_sub_ target levels) with microbubbles in circulation (rabbit #3, mean target location = [-4,0,-31] mm in array coordinates). Data is shown from both the calibration (time = 0-42 s, *τ* = 3 ms) and fixed-pressure (time = 50-169 s, *τ* = 10 ms) sonication stages. The microbubble infusion window (time = 0-90 s) is indicated by the shaded regions. Color-coded dashed lines in (B) represent SPTA source field intensity data from the corresponding baseline sonication without microbubbles in circulation. (**C**) Maximum intensity projection (MIP) contour images of ultrafast 3D PCI data (*τ* = 100 µs) for the burst during which spatially-coherent subharmonic activity was detected for each grid point during calibration ('trigger burst', data from each grid point is self-normalized), as well as for sonication-aggregate data (each grid point normalized to the 150% *p*_sub_ target level data). Linear contours are displayed at 10% intervals starting at 20%. The dotted gray lines overlaid on the coronal and sagittal MIPs for the sonication-aggregate data indicate the superior-inferior coordinate of the axial target plane. Scale bars indicate 2 mm. (**D**) Sonication-averaged power spectrum of the unfiltered beamformed signal at the location of SPTA source field intensity for each target level, normalized to the power spectral density at the transmit frequency (***f***_0_ = 612 kHz) in the 150% *p*_sub_ target level data and plotted on a decibel scale. (**E**) *In-vivo* SPTPN pressure subharmonic threshold values (cohort #1) stratified based on target level, target location, and microbubble dose. The number of samples per group is indicated by *n*, and * denotes a statistically significant difference between groups (p < 0.05). Error bars represent one SD.

An example of multi-point 3D subharmonic calibration with microbubbles in circulation is shown in **Figure 2**. Starting simultaneously with an intravenous microbubble infusion, the SPTPN pressure level (**Figure 2A**) was increased at each grid point sequentially until spatially-coherent subharmonic microbubble activity was detected, which typically coincided with an increase in the spatial-peak temporal-average (SPTA) source field intensity of the corresponding 3D PCI data relative to both the previous burst and to baseline sonications without circulating microbubbles (**Figure 2B**). In this example, once subharmonic activity was detected at all four grid points (**Figure 2C**), the SPTPN pressure applied at each grid point was fixed to a different percentage of the corresponding target's subharmonic pressure threshold (*p*_sub_) for the remainder of the sonication (*i.e.*, 0%, 50%, 100%, or 150% *p*_sub_). Sonication-aggregate ultrafast 3D PCI data revealed spatially-coherent microbubble activity throughout the exposures performed at 100% *p*_sub_ and 150% *p*_sub_ (**Figure 2C**), the spatial extent of which increased with increasing exposure level and with signal content extending into the near-field region (*i.e.*, ~5 mm proximal to the array from the target plane) at the 150% *p*_sub_ target level. The sonication-averaged power spectra of the beamformed signals at the location of SPTA source field intensity contained pronounced subharmonic and wideband spectral content at 100% *p*_sub_ and 150% *p*_sub_ (**Figure 2D**), indicating the presence of sustained inertial cavitation activity at these exposure levels.

Spatially-coherent subharmonic activity was successfully detected *in vivo* via 3D PCI during the calibration stage of all multi-point sonications performed with microbubbles in circulation (**Figure 2E**). No statistical differences were found in the mean SPTPN pressure subharmonic threshold when the grid points were stratified based on the exposure level (p = 0.51). The posterior-most grid points required greater pressures to initiate subharmonic activity than their anterior counterparts (left-posterior vs. right-anterior: p = 0.04, left-posterior vs. left-anterior: p = 0.04). The mean *in-situ* subharmonic pressure threshold determined intraoperatively during the multi-point calibration stage in this study (animal cohort #1: *p*_sub_ = 0.67 ± 0.08 MPa, 0.20 ml/kg Definity^TM^ microbubbles) was not significantly different (p = 0.64) from that obtained in previous work with a similar experimental setup but an order of magnitude lower microbubble dose (*p*_sub_ = 0.66 ± 0.12 MPa, 0.02 ml/kg Definity^TM^ microbubbles 47). A summary of the PCI data from all multi-point calibration sonications *in vivo* is provided in **Table 1**.

**Figure S1A** shows SPTA source field intensity data during the fixed-pressure sonication stage for each target location in each animal from cohort #1, along with an animal-wise average for each target level. Signal patterns consistent with microbubble infusion kinetics can be seen for the exposures performed at 100%* p*_sub_ and 150%* p*_sub_, whereas at lower target levels (*i.e.*, 50% *p*_sub_ and 0% *p*_sub_) the signal remained within the noise floor. No evidence of microbubble activity was detected during any of the exposures performed at either 50% *p*_sub_ or 0% *p*_sub_ (*i.e.*, 9 animals × 2 targets/animal × 120 bursts/target = 2,160 bursts total).

### Megahertz-rate volumetric imaging uncovers spatiotemporal microbubble dynamics *in vivo*

**Figure 3:**
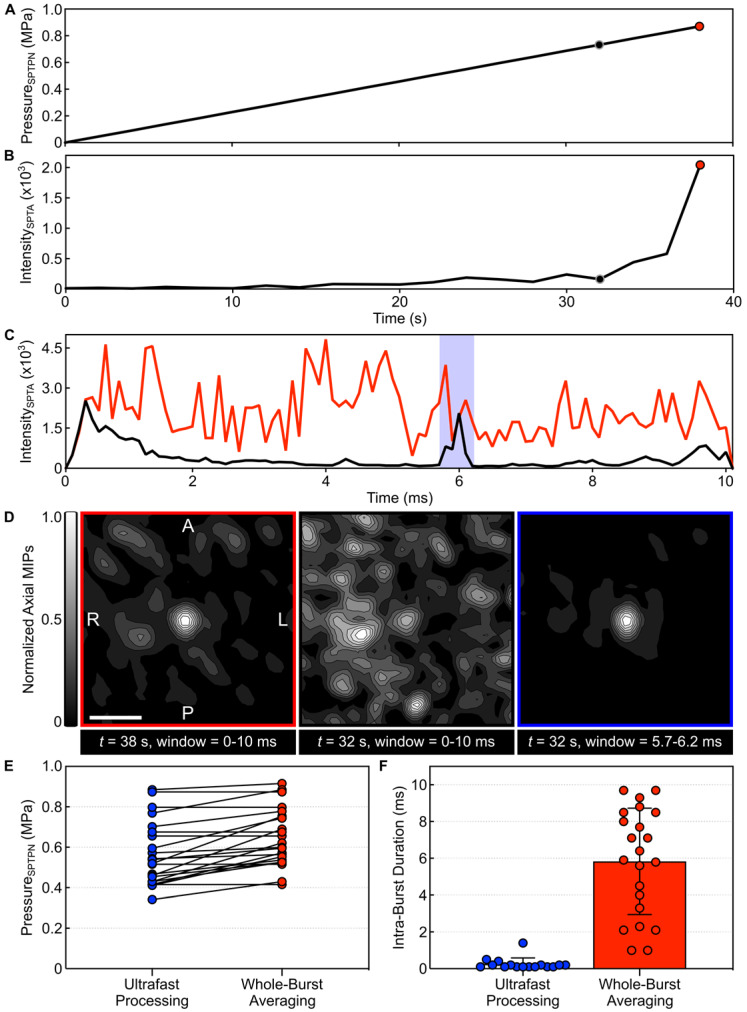
** Ultrafast 3D acoustic imaging uncovers spatiotemporal microbubble dynamics *in vivo*.** (**A**) Estimated *in-situ* SPTPN pressure and (**B**) SPTA source field intensity (*τ* = 10 ms) as a function of time during a single-point calibration sonication with microbubbles in circulation (rabbit #10, target location = [-5,1,-9] mm in array coordinates). (**C**) SPTA source field intensity as a function of time throughout the 10 ms burst length for the bursts 32 s (SPTPN pressure = 0.73 MPa) and 38 s (SPTPN pressure = 0.87 MPa) into the sonication (*τ* = 100 µs). (**D**) Normalized axial MIP contour images of 3D PCI data for the bursts 32 s and 38 s into the sonication generated via whole-burst temporal averaging (*τ* = 10 ms), and for the burst 32 s into the sonication with a 500 μs beamforming window denoted by the shaded blue region in (C). The axial imaging FOV is centered on the target location. Scale bar indicates 5 mm. (**E**) *In-vivo* SPTPN pressure subharmonic threshold values (cohort #2) obtained retrospectively via ultrafast processing (*τ* = 100 µs) and intraoperatively via whole-burst temporal averaging (*τ* = 10 ms). (**F**) Intra-burst microbubble activity duration within the first burst of the calibration sonication in which subharmonic activity is detected using both ultrafast processing (*τ* = 100 µs) and whole-burst temporal averaging (*τ* = 10 ms). Data in (E,F) corresponds to a total of 22 calibration sonications across all 8 animals in cohort #2.

Spatially-coherent microbubble activity was detected successfully *in vivo* via 3D PCI with integration window lengths (*τ*) as short as 1 μs, corresponding to imaging volume rates of up to 1 MHz (**Movie S1**). An example of short-time analysis of a single-point calibration sonication is shown in **Figure 3**. The SPTPN pressure level (**Figure 3A**) was increased burst-by-burst until spatially-coherent subharmonic microbubble activity was detected intraoperatively via whole-burst temporal average processing (*i.e.*,* τ* = 10 ms), which occurred during the burst 38 s into the sonication (**Figure 3B**). Retrospective short-time analysis (**Figure 3C**) with an imaging volume rate of 10 kHz (*i.e.*,* τ* = 100 μs) revealed a short duration (~0.5 ms of the 10 ms burst length) of spatially-coherent subharmonic activity embedded within the burst 32 s into the sonication that was missed via whole-burst temporal-averaging (**Figure 3D**).

Examining all calibration sonications from cohort #2, retrospective short-time analysis with an imaging volume rate of 10 kHz frequently (*i.e.*, in 73% [16/22] of sonications) revealed sporadic subharmonic activity at pressure levels below the threshold determined intraoperatively via whole-burst temporal-average processing (**Figure 3E**). The subharmonic SPTPN pressure threshold determined retrospectively in this cohort (*p*_sub_ = 0.57 ± 0.16 MPa) was 87 ± 12% of that obtained intraoperatively (*p*_sub_ = 0.65 ± 0.14 MPa). The mean duration of intra-burst microbubble activity (**Figure 3F**) observed within the burst of the calibration stage during which subharmonic activity was first detected retrospectively with ultrafast processing (0.3 ± 0.5 ms) was over an order of magnitude shorter than for the burst identified intraoperatively via temporal averaging (6 ± 3 ms).

**Figure S1B** shows SPTA source field intensity data throughout the 10 ms burst length for each target location in each animal from cohort #1 (*i.e.*, fixed-pressure sonication stage only), along with an animal-wise average for each target level. Evidence of microbubble activity persisting throughout the entire 10 ms burst length can be seen for the exposures performed at 100%* p*_sub_ and 150%* p*_sub_, whereas at lower target levels (*i.e.*, 50% *p*_sub_ and 0% *p*_sub_) the signal remained within the noise floor.

### Sonication-aggregate ultrafast 3D microbubble imaging data predicts tissue damage distributions during nonthermal brain ablation

**Figure 4:**
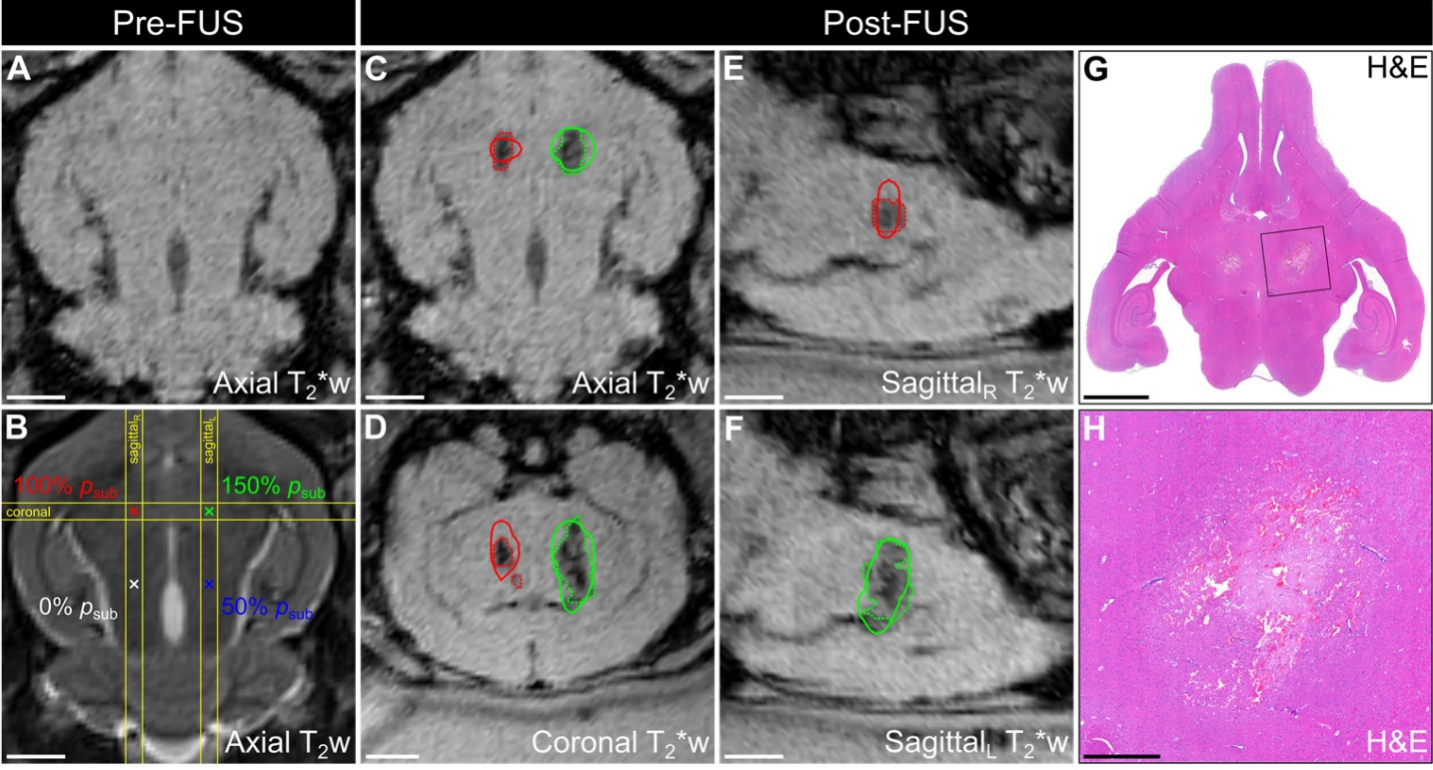
** Spatial correlation of ultrafast 3D microbubble imaging data with volumetric FUS-induced brain tissue damage.** Axial (**A**) T_2_*w and (**B**) T_2_w MRI data acquired pre-sonication (rabbit #5, mean target location = [1,8,-23] mm in array coordinates, point spacing = 6 mm). Color-coded target locations and corresponding target levels are overlaid in (B). (**C**) Axial, (**D**) coronal, and (**E,F**) sagittal T_2_*w MRI data acquired immediately post-sonication. The coronal and sagittal slice volumes are indicated in (B) by the yellow lines. Segmented regions of T_2_*w MRI signal hypointensity induced by the exposures (dotted lines) and corresponding sonication-aggregate ultrafast PCI contour data at the operating threshold that maximizes the global PR curve F_1_-score of the MRI_3D_ dataset (solid lines) are overlaid and are color-coded by target level (green = 150% *p*_sub_, red = 100% *p*_sub_, blue = 50% *p*_sub_, black = 0% *p*_sub_). Scale bars on the MRI data indicate 5 mm. (**G**) Axial H&E stained tissue section corresponding to the axial MRI plane (sacrifice time point = 48 h post-sonication). (**H**) Magnified view of the left-anterior grid point (150% *p*_sub_ target level). Scale bars on the H&E data in (G) and (H) indicate 5 mm and 1 mm, respectively.

Multimodal imaging data from a representative animal in cohort #1 is depicted in **Figure 4**. Baseline MRI data were acquired pre-sonication for targeting and to assist with the identification of any abnormalities induced by the exposures (**Figure 4A,B**). In this animal, T_2_*-weighted (T_2_*w) MRI acquired immediately post-sonication displayed regions of signal hypointensity induced by the exposures at 100% *p*_sub_ and 150% *p*_sub_, but not at lower target levels (*i.e.*, 50% *p*_sub_ and 0% *p*_sub_), and the spatial extent of the FUS-induced hypointensities increased with increasing exposure level (**Figure 4C-F**). Ultrafast microbubble imaging data revealed spatially-coherent microbubble activity throughout the exposures at 100% *p*_sub_ and 150% *p*_sub_, and the sonication-aggregate 3D PCI data co-localized well with regions of FUS-induced T_2_*w MRI signal hypointensity (**Figure 4C-F**). The centroids of the binary tissue damage volumes predicted via 3D PCI were located within a half-millimeter of those measured using T_2_*w MRI (100% *p*_sub_: 0.4 mm, 150% *p*_sub_: 0.2 mm, **Table 2**). Hematoxylin-eosin (H&E)-stained tissue sections from this animal (**Figure 4G-H**) associated the T_2_*w MRI signal hypointensities with the presence of red blood cells (RBCs) scattered throughout focal regions of tissue necrosis 48 h post-sonication.

Cross-sectional imaging data from the focal plane of each animal in cohort #1 is provided in **Figure S2**. Regions of T_2_*w MRI signal hypointensity were induced in the focal zone of all 9 targets exposed at 150% *p*_sub_ and in 7 of 9 targets exposed at 100% *p*_sub_ (*i.e.*, rabbits #1-7). In contrast, only 1 of 18 locations exposed at either 50% *p*_sub_ or 0% *p*_sub_ was associated with focal T_2_*w abnormalities post-sonication (*i.e.*, rabbit #3, left-anterior target location, 0% *p*_sub_); a small hypointense volume was detected at this target location immediately post-sonication (*i.e.*, 3.1 mm^3^), which corresponded to the grid point with the largest SPTA source field intensity during the calibration sonication stage in that animal (**Figure 2B**). The spatial extent of the FUS-induced T_2_*w MRI signal hypointensities were found to increase with increasing exposure level in each of the 9 animals in cohort #1. Coronal T_2_*w MRI scans revealed signal hypointensities in the pre-focal region (*i.e.*, impingent upon or dorsal to the third and lateral ventricles, extending into the superior cortex) in 7 of 9 targets exposed at 150% *p*_sub_ (*i.e.*, rabbits #1-7), and in 2 of 9 targets exposed at 100% *p*_sub_ (*i.e.*, rabbits #1-2). Sonication-aggregate ultrafast 3D PCI data co-localized well with regions of FUS-induced T_2_*w MRI signal hypointensity, including the targets at which near-field effects were observed. Across all targets, the centroids of the binary damage volumes predicted via sonication-aggregate 3D PCI were located within a millimeter (0.9 ± 0.6 mm, mean ± SD) of those measured using T_2_*w MRI (**Table 2**), corresponding to a spatial accuracy on the order of the MRI voxel size employed. In the animals sacrificed 24 h and 48 h post-sonication (*i.e.*, rabbits #1-7), H&E histology revealed RBCs scattered throughout focal regions of tissue necrosis at the targets exposed at 100% *p*_sub_ and 150% *p*_sub_, as well as small zones of tissue damage at the lower target levels (*i.e.*, 50% *p*_sub_ or 0% *p*_sub_) that were not evident on T_2_*w MRI. Glial scars were present at the 150% *p*_sub_ target level in both animals sacrificed 8 d post-sonication (*i.e.*, rabbits #8-9).

**Figure S3** presents multi-sequence MRI data acquired both immediately- and 24 h post-sonication for rabbit #5, the same animal displayed in **Figure 4**. Contrast-enhanced T_1_-weighted (T_1_w) MRI signal enhancement was detected at all four targets immediately post-sonication, indicative of increased levels of BBB permeability, which persisted at the three highest target levels (*i.e.*, 50%, 100%, and 150% *p*_sub_) at the 24 h time point. Pre-focal regions of increased BBB permeability were found along the FUS beam paths, particularly for exposures at 100% *p*_sub_ and 150% *p*_sub_. Substantial edema and brain swelling was present on T_2_-weighted (T_2_w) MRI at the 24 h time point at the three highest target levels investigated (*i.e.*, 50%, 100%, and 150% *p*_sub_), whereas comparatively subtle changes were evident immediately post-sonication. The volumes of FUS-induced T_2_*w hypointensity present at the 24 h time point were similar to those measured via scans acquired immediately post-sonication, however, the mean T_2_*w MRI signal magnitude within these regions was increased at the later time point (*i.e.*, less hypointense relative to the surrounding normal tissue).

**Figure 5:**
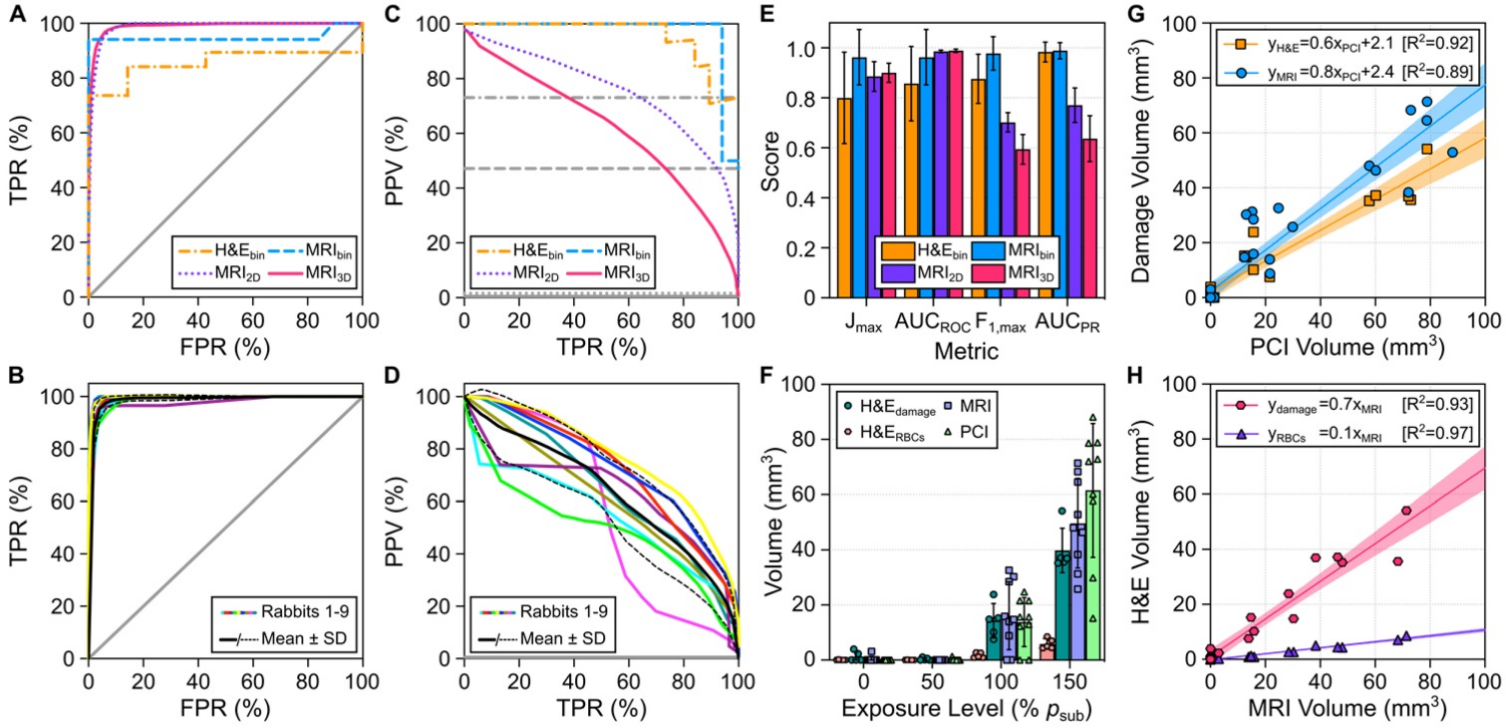
** Predictive capability of ultrafast 3D microbubble imaging data to detect FUS-induced brain tissue damage distributions.** (**A**) Global ROC curves for binary classification of tissue damage on both MRI (MRI_bin_) and H&E (H&E_bin_), as well as 2D pixel-wise (MRI_2D_) and 3D voxel-wise (MRI_3D_) classification of tissue damage on MRI acquired immediately post-sonication via sonication-aggregate ultrafast 3D PCI data. (**B**) Animal-wise ROC curves for the MRI_3D_ dataset, along with an average ROC curve across all animals in cohort #1. (**C**) and (**D**) present the PR counterpart data to (A) and (B), respectively. In (A-D), the gray lines denote the ROC/PR curves for classifiers with random performance for a specific dataset (solid = MRI_3D_, dotted = MRI_2D_, dashed = MRI_bin,_ dash-dotted = H&E_bin_). (A-D) TPR = True Positive Rate, FPR = False Positive Rate, PPV = Positive Predictive Value. (**E**) Metrics used for evaluating ROC (*J*_max_, AUC_ROC_) and PR (F_1,max_, AUC_PR_) curves for each classification dataset (animal-wise mean ± SD). *J*_max_ and F_1,max_ correspond to the maximum values of *J* and F_1_ obtained from the ROC and PR curves, correspondingly. (**F**) Segmented tissue damage volumes (H&E damage, H&E RBCs, T_2_*w MRI hypointense) and volumes predicted by 3D PCI stratified based on exposure level (mean ± SD). (**G**) Linear regression between the segmented tissue damage volume (T_2_*w MRI hypointense, H&E damage) and the volume predicted by 3D PCI. (**H**) Linear regression between the H&E segmented volume (H&E damage, H&E RBCs) and the T_2_*w MRI hypointense volume. In (G,H) the lines of best fit and coefficient of determination (R^2^) values are listed as insets, and the shaded regions represent the 95% confidence bands. The segmented H&E volume data in (F-H) correspond to animals sacrificed at the 48 h time point (rabbits #2-6).

The predictive capability of sonication-aggregate ultrafast 3D source field intensity distributions for detecting tissue damage induced during microbubble-mediated nonthermal ablation was assessed using receiver operating characteristic (ROC) and precision-recall (PR) analyses (**Figure 5A-E**). ROC and PR curves were generated for binary (MRI_bin_, H&E_bin_), 2D (MRI_2D_), and 3D (MRI_3D_) classifications of tissue damage. ROC analysis of our data suggested near-perfect tissue damage classification throughout the brain (*e.g.*, AUC_ROC_ = 0.99 ± 0.01 for both MRI_2D_ and MRI_3D_ datasets), whereas the corresponding PR analysis provided a more realistic depiction of classifier performance in terms of predicting the spatial distribution of the damaged region (*e.g.*, MRI_2D_ dataset: AUC_PR_ = 0.77 ± 0.07, MRI_3D_ dataset: AUC_PR_ = 0.64 ± 0.09). Examining the PR curves (**Figure 5C-D**), the best performance (*i.e.*, highest F_1_-score and AUC_PR_ values) was obtained for the case of binary classification (*i.e.*, MRI_bin_ dataset: F_1_-score = 0.98 ± 0.07, H&E_bin_ dataset: F_1_-score = 0.88 ± 0.10). Sonication-aggregate 3D PCI data were capable of predicting spatial distributions of T_2_*w MRI signal hypointensity both in 2D within the axial focal plane (*i.e.*, MRI_2D_ dataset: F_1_-score = 0.70 ± 0.04) and in 3D within the entire rabbit brain (*i.e.*, MRI_3D_ dataset: F_1_-score = 0.60 ± 0.06). Both the F_1_-score and AUC_PR_ values obtained from the PR curve corresponding to the 2D classification of tissue damage were larger than those resulting from the counterpart 3D analysis. The aforementioned trends are summarized in **Figure 5E**, which plots different metrics used for quantitative evaluation of the ROC and PR curves for each classification dataset. Ultrafast microbubble imaging data provided superior predictive capability than that obtained via conventional temporal average processing (**Figure S4**).

The tissue damage volumes predicted via sonication-aggregate 3D PCI with the operating threshold that maximized the F_1_-score of the global PR curve were compared with the damage volumes measured post-sonication via MRI and H&E (**Figure 5F-H**). The average tissue damage volumes assessed via both MRI and H&E were found to increase with increasing exposure level, and the volume predicted by sonication-aggregate 3D PCI increased concomitantly (**Figure 5F**). H&E histology revealed small zones of RBC extravasations (range = 0.001-0.198 mm^3^) and overt tissue damage (range = 0.1-3.9 mm^3^) resulting from the exposures at 50% *p*_sub_ and 0% *p*_sub_ that were not evident on T_2_*w MRI (**Figure 5F**). On a target-by-target basis, the tissue damage volumes measured with both MRI and H&E were found to correlate linearly with the volume predicted by sonication-aggregate 3D PCI (**Figure 5G**), and the volumetric H&E segmentations (*i.e.*, total overt tissue damage, RBC extravasations only) both correlated linearly with the volume of signal hypointensity on T_2_*w MRI (**Figure 5H**).

### Under-sampling of acoustic emissions can reduce the predictive capability of microbubble imaging data

**Figure 6:**
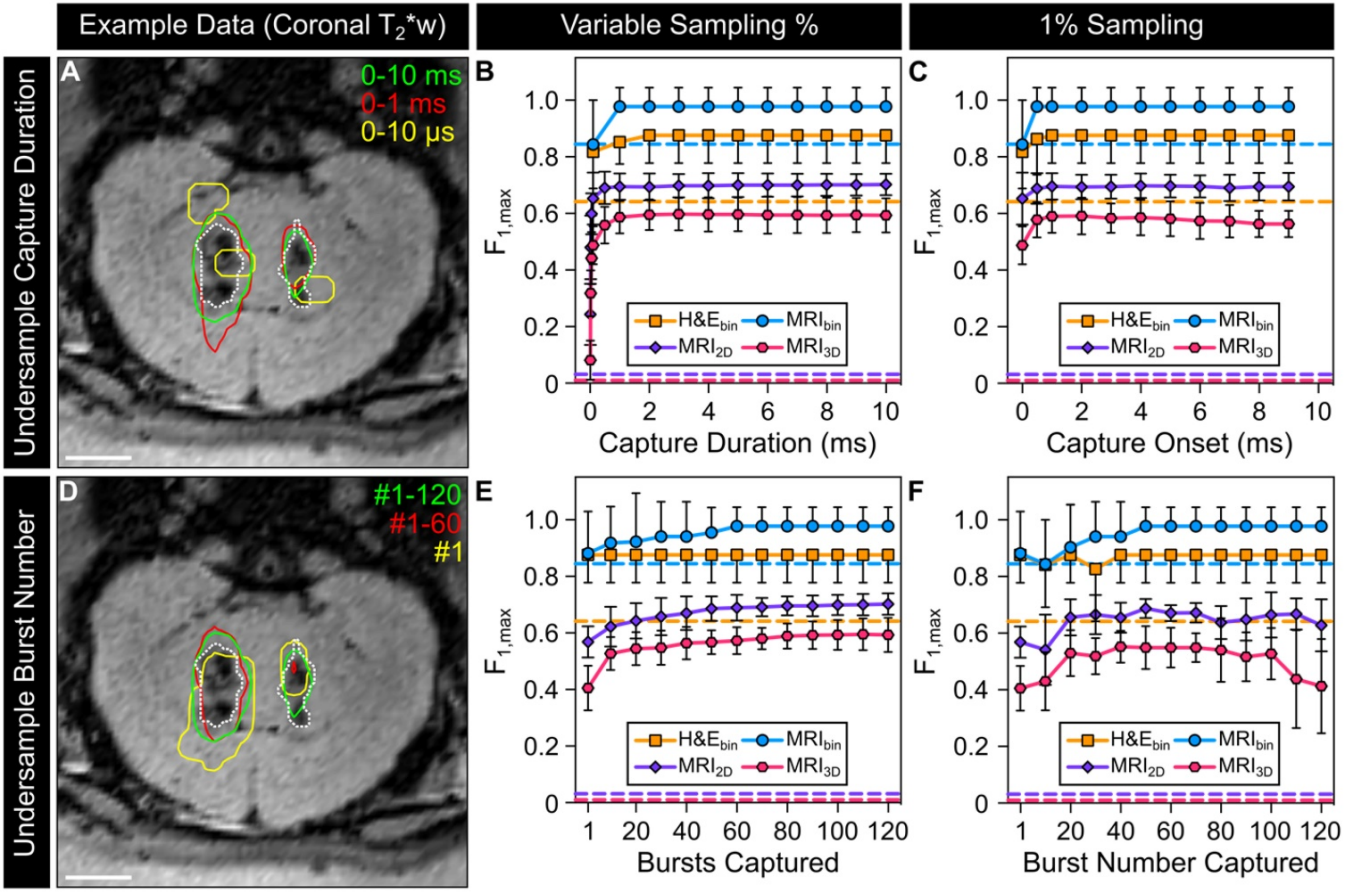
** Effects of undersampling acoustic emissions on the predictive capability of microbubble imaging data.** (**A**) Example of undersampling the 10 ms burst length (capture duration per burst < 10 ms, number of bursts captured = 120). Sonication-aggregate ultrafast PCI contour data at the operating threshold that maximizes the global PR curve F_1_-score of the MRI_3D_ dataset are overlaid (solid lines) and are color-coded by capture window (green = 0-10 ms, red = 0-1 ms, yellow = 0-10 µs). (**B**) Maximum F_1_-score (F_1,max_) as a function of the capture duration per burst (capture onset time = start of 10 ms burst). (**C**) F_1,max_ as a function of the capture onset time within the 10 ms burst (capture duration = 100 µs). (**D**) Example of undersampling the 120 burst exposure duration (number of bursts captured < 120, capture duration per burst = 10 ms). Sonication-aggregate ultrafast PCI data at the operating threshold that maximizes the global PR curve F_1_-score of the MRI_3D_ dataset are overlaid (solid lines) and are color-coded by the burst numbers captured (green = #1-120, red = #1-60, yellow = #1). (**E**) F_1,max_ as a function of the number of bursts captured (capture onset time = start of 120 burst exposure). (**F**) F_1,max_ as a function of the single burst captured within the 120 burst exposure. In (B,C,E,F) animal-wise F_1_-scores (mean ± SD) are provided for binary classification of tissue damage on both MRI (MRI_bin_) and H&E (H&E_bin_), as well as 2D pixel-wise (MRI_2D_) and 3D voxel-wise (MRI_3D_) classification of tissue damage on MRI. F_1_-scores were calculated independently for each animal using the operating threshold that maximizes the global PR curve F_1_-score. The color-coded dashed lines denote the F_1_-score of classifiers with random performance for each dataset. One-way error bars are plotted for the binary classification data for visualization purposes. In (A,D) the coronal imaging plane intersecting the 100% *p*_sub_ (target location = [-6,8,-21] mm in array coordinates) and 150% *p*_sub_ (target location = [0,8,-21] mm in array coordinates) targets is shown and segmented regions of T_2_*w MRI signal hypointensity induced by the exposures are overlaid (dotted white lines, rabbit #2). Scale bars indicate 5 mm.

The impact of temporally under-sampling acoustic emissions on the predictive capability of the resulting sonication-aggregate ultrafast 3D PCI data was investigated retrospectively for different sampling strategies (**Figure 6**). **Figure 6A** provides example data for the scenario in which the capture duration for each burst within the sonication is less than the 10 ms burst length. Under-sampling of the capture duration introduces regions of false positive and false negative signal content in the resulting sonication-aggregate 3D PCI data, particularly for short capture durations. For each classification dataset investigated (*i.e.*, H&E_bin_, MRI_bin_, MRI_2D_, and MRI_3D_), the F_1_-score increased with increasing capture duration until approximately 1-2 ms, with no benefit obtained for longer capture times (**Figure 6B**). For very short capture durations (*e.g.*, beamforming the first 10 μs of the 10 ms burst length, or 0.1% capture duration), the F_1_-scores for the MRI_2D_ and MRI_3D_ classification datasets approached the values obtained from classifiers with random performance (**Figure 6B**). With a fixed capture duration of 1% (*i.e.*, 100 μs of the 10 ms burst length), the highest F_1_-scores for the MRI_3D_ classification dataset were obtained for capture onsets of 1 ms and 2 ms, whereas beamforming of the acoustic emissions data from the beginning of the burst provided the worst performance of all capture onsets investigated (**Figure 6C**).

**Figure 6D** provides example data for the scenario in which the total number of bursts captured within the sonication is fewer than the 120 burst total. Under-sampling the number of bursts was also found to introduce false positive and false negative signal content in the resulting sonication-aggregate 3D PCI data. For the MRI_2D_ and MRI_3D_ classification datasets, the F_1_-scores increased monotonically as the number of bursts captured was increased towards the 120 burst total (**Figure 6E**). For the case in which only a single burst was captured within the sonication (*i.e.*, ~1% sampling) the trends of the F_1_-score as a function of the burst number captured were not generalizable across the different classification datasets, though for both the MRI_2D_ and MRI_3D_ datasets the highest F_1_-scores were obtained when sampling within the middle two thirds of the sonication (*i.e.*, burst ~ #20-100, **Figure 6F**).

## Discussion

Microbubble-mediated FUS therapies represent a potentially disruptive treatment approach for a variety of CNS diseases and other medical indications. However, given the associated risks their widespread clinical adoption will rely on the ability to monitor and control the induced bubble activity in real-time throughout the exposures, to ensure delivery of the desired bioeffect distribution(s) without any adverse events. Here, it has been demonstrated for the first time that ultrafast 3D microbubble imaging data can predict the tissue damage volume distributions induced during nonthermal brain ablation, which represents a critical step toward achieving full volumetric control of treatment response to microbubble-mediated FUS therapies.

Microbubble-mediated nonthermal ablation, also referred to as 'mechanical ablation', 'antivascular ultrasound therapy', or 'vascular disruption therapy' 24-35, is an innovative technique for non-invasively ablating brain tissue structures throughout the skull cavity. This emerging approach has the potential to increase the efficiency of current clinical thermoablative FUS brain procedures 10-14 and enable the treatment of peripheral tumors and disorders for which off-central (*e.g.*, epilepsy 15) or near-skull base (*e.g.*, trigeminal neuralgia 16) targets are required. The ability to predict the volumetric distribution of tissue damage induced during nonthermal ablation represents a major step towards clinical translation of this non-invasive surgical approach, however, additional work is warranted prior to initial human testing. Investigations into 'optimal' treatment parameters that minimize unwanted bioeffects are needed, which includes factors related to both the ultrasound pulsing scheme as well as the microbubble dosing and administration protocols. For instance, it would be desirable to induce tissue necrosis directly while minimizing both RBC extravasation production in the focal region and increased BBB permeability along the FUS beam path. In addition, the achievable 'treatment envelope' should be evaluated through experiments in large animal models with *ex-vivo* human skullcaps 49 to determine the feasibility of treating peripheral targets within the human skull cavity.

The current study builds upon our initial work on 3D subharmonic imaging-based FUS exposure level calibration 47 by enabling multiple target locations to be calibrated separately within the same sonication (**Figure 2**). Although multi-point exposure level calibration has been demonstrated previously using single-element detector cavitation feedback 63, to the best of our knowledge this is the first study to demonstrate multi-point calibration via 3D microbubble imaging. Microbubble dose did not influence the SPTPN pressure level required to detect subharmonic activity when comparing two groups that spanned an order of magnitude in dose (*i.e.*, 0.02 vs. 0.20 ml/kg Definity^TM^ microbubbles), a result that is in line with the findings from a previous pre-clinical study in rodents with the same FUS pulsing scheme (*i.e.*, 0.01 vs. 0.10 ml/kg Definity^TM^ microbubbles 64). This result further demonstrates the robustness of calibrating exposure levels during cavitation-mediated FUS therapies based on indicators of *in-situ* microbubble activity provided via acoustic emissions analysis, at least across the dose range investigated herein.

In a previous study of sonothrombolysis *in vitro* without exogenous cavitation nuclei, our group demonstrated that short-time analysis of 3D PCI data over microsecond timescales uncovered details regarding the evolution of cavitation activity that were hidden when temporal averaging was performed over the duration of FUS on-time 65. Here, the first *in-vivo* application of ultrafast 3D microbubble imaging using PCI is reported. The volume rates achieved with this approach (*i.e.*, ~MHz) are several orders of magnitude faster than those obtainable with conventional 3D ultrasound contrast or B-mode imaging (*i.e.*, ~10 Hz), or even more advanced plane wave methods (*i.e.*, ~10 kHz) 66. In this study, retrospective high volume rate microbubble imaging during the calibration sonication stage frequently revealed sporadic subharmonic activity at pressure levels below the thresholds determined intraoperatively via whole-burst temporal average processing (**Figure 3**). This is a pertinent finding from an exposure level calibration perspective, as more reliable estimates of the pressures required to initiate subharmonic activity *in vivo* could potentially be exploited to produce more consistent cavitation-mediated FUS treatment outcomes in the future. Moreover, ultrafast processing was shown to improve the predictive capability of sonication-aggregate 3D PCI data for detecting FUS-induced tissue damage volume distributions relative to conventional temporal averaging approaches (**Figure S4**). However, given the increased computational complexity associated with ultrafast processing techniques, the use of acceleration methods will be needed to enable real-time implementations in practice.

Although good qualitative (**Figure 4** and **Figure S2**) and quantitative (**Figure 5, Table 2**) agreement was obtained between ultrafast 3D microbubble imaging data and the spatial distribution of FUS-induced tissue damage, both the signal acquisition and processing pipelines stand to be improved upon in future work. For instance, as acoustic emissions data were acquired using narrow-band transducer elements tuned to the subharmonic of the driving frequency, only a relatively small bandwidth of the total microbubble emission spectra (*i.e.*, 200-400 kHz) could be monitored reliably. Although this approach allowed sufficient detection of acoustic emissions to map volumetric microbubble activity *in vivo* over microsecond timescales (**Movie S1**), the reduced levels of receiver sensitivity at other frequency bands of interest (*e.g.*, harmonics 38, ultraharmonics 46) may have hindered the predictive capability of the resulting PCI data. Wideband sensor arrays 67 could be employed to increase the detectable bandwidth of the microbubble emission spectra, however, the reduced levels of sensitivity associated with piezoelectric polymers 45 combined with the increased attenuation of human skull at higher source frequencies 43 may pose additional challenges in a clinical setting. From a signal processing perspective, the techniques employed here to combine PCI data within each burst and from burst-to-burst to generate sonication-aggregate data are nascent, principally because appropriate benchmark experimental data (*i.e.*, 3D PCI with acoustic emissions captured over total duration of FUS on-time, together with co-registered volumetric bioeffect distributions) was not previously available in the literature. Investigations into alternative methods for generating sonication-aggregate PCI data, both in the context of microbubble-mediated nonthermal ablation as well as other cavitation-mediated therapies, are warranted and may improve the predictive capability for detecting FUS-induced bioeffect distributions.

Despite having calibrating the FUS exposures at each target location based on the pressure required to detect subharmonic microbubble activity *in vivo*, substantial variability was observed in the resulting tissue damage volumes for a fixed target level (**Figure 5F**). In this work the FUS exposures became open-loop following subharmonic calibration (*i.e.*, during the fixed-pressure sonication stage). As a result, any changes experienced by the microbubbles during the sonications (*e.g.*, growth or shrinkage from being in the vasculature) were not accounted for, which may have contributed to the large variability observed in the spatial extent of FUS-induced bioeffects. This variability could potentially be reduced in the future through the incorporation of 3D microbubble imaging within a closed-loop exposure control framework 41,48. Nevertheless, the tissue damage volumes measured post-sonication with MRI and histology were both linearly correlated with the volumes predicted via sonication-aggregate 3D PCI data (**Figure 5G**). Moreover, the tissue damage volumes estimated via T_2_*w MRI were linearly correlated with the volume of tissue damage 48 h post-sonication (**Figure 5H**), demonstrating the utility of T_2_*w MRI as a biomarker during microbubble-mediated nonthermal ablation procedures.

Another finding from this work is that the analysis method chosen to evaluate the predictive capability of PCI data to detect FUS bioeffect distributions can have a profound impact on the perception of the obtained results. Studies conducted to date within the therapeutic ultrasound community have exclusively employed ROC analysis for this purpose 68-70. However, it has been well documented in other fields of study that PR analysis can be more informative than ROC analysis when the dataset under investigation has a class imbalance, particularly when one class is of preferential relevance 71. In the context of predicting FUS-induced tissue damage, the positive (P) class (*i.e.*, damaged region) is generally of greater interest than the negative (N) class (*i.e.*, undamaged region), and the datasets can be substantially unbalanced (*e.g.*, global MRI_3D_ dataset: P/(P+N) = 0.5%, **Table 3**) although information regarding the class balance is rarely reported in the literature. The disparity between the perceived outcomes of ROC and PR analysis for the case of unbalanced datasets was demonstrated directly in this study (**Figure 5**); ROC analysis provided a highly reliable prediction of tissue damage classification within the entire brain volume but drastically inflated the ability of sonication-aggregate ultrafast 3D PCI data to classify the spatial distribution of the damaged region, whereas PR analysis provided a more realistic depiction of the latter. Therefore, appropriate care needs to be taken when selecting methods for evaluating the predictive capability of PCI data to detect FUS bioeffect distributions, and information regarding the class balance of the dataset(s) under investigation should always be reported transparently.

Studies conducted to date that have correlated PCI data with FUS-induced bioeffects have not acquired acoustic emissions data over the total duration of ultrasound on-time due to hardware limitations 50,55-57,68-70. The dataset acquired during the current study therefore afforded the opportunity to investigate, for the first time, how temporal under-sampling of acoustic emissions affects the predictive capability of the resulting PCI data (**Figure 6**). Retrospective under-sampling introduced regions of false positive and false negative signal content in the resulting sonication-aggregate 3D PCI data, the extent of which depended on the temporal sampling paradigm that was carried out. For the traditional approach in which signal acquisition commences at the beginning of the burst 50,55-57,68-70, the F_1_-score obtained from fully-sampled acoustic emissions data was reached with a capture duration of 1-2 ms, with no appreciable benefit obtained from longer capture durations. This result suggests that the information contained within the first 1-2 ms of each burst is sufficient to predict how the target tissue will respond to the full 10 ms burst length, at least within the current dataset. In fact, the F_1_-score obtained from the fully-sampled case could be recovered with a capture duration per burst of only 100 μs (*i.e.*, 1% of the 10 ms burst length), depending on which portion of the burst was acquired. For the case of 1% sampling, capture onset times of 1 ms and 2 ms provided the best performance, whereas the case in which signal acquisition was performed at the beginning of the burst resulted in the worst outcome of all capture onsets investigated. In contrast, when the entire burst length was captured per burst but only a single burst was captured (*i.e.*, 1/120 = 0.8% sampling) the F_1_-score obtained from the fully-sampled case could not be recovered regardless of which burst was captured. Taken together, these findings suggest that the temporal sampling strategies employed in previous studies (*i.e.*, 0.01-6% sampling at the start of each 7-10 ms burst 50,55-57) may have resulted in sub-optimal correlations between PCI data and FUS bioeffect distributions.

There are additional limitations to the current study apart from those already discussed. For instance, although the low duty cycle exposures employed were not expected to induce substantial bulk tissue temperature elevations 17,21, focal temperature changes could not be monitored directly as the prototype FUS brain system was not MRI-compatible. Sub-ablative focal temperature elevations (*e.g.*, up to 10.0 ± 7.3 ^o^C in Ref. 26) have been reported during microbubble-enhanced FUS ablation 25,26,30,31, and such thermal effects may have contributed to the biological outcomes reported in these studies. Future work will aim to evaluate the extent of focal heating generated from our FUS treatment paradigm (*e.g.*, via intraoperative MR-thermometry 7), to further elucidate the underlying mechanisms at play. Another limitation is that a subset of our study was performed on craniotomized animals (cohort #1) to allow delivery of sufficient acoustic pressure levels within the brain using our prototype FUS system, which does not reflect the clinical scenario in which an intervening skull would be present between the array and target tissue. Nevertheless, microbubble-mediated ultrasonic brain therapy delivered through the intact skull is feasible in humans using commercial FUS systems 19-22. Similarly, trans-human skull detection of microbubble cavitation emissions via single-element detectors 20,21 and 3D PCI via multi-element detector arrays 51,59 have been demonstrated in clinical and pre-clinical studies, respectively. Although previous work has shown that transcranial aberrations can be mitigated on receive using non-invasive compensation techniques 58-60, future investigations into how these distortions impact the ability of 3D PCI data to predict FUS bioeffect distributions in the brain are warranted.

Beyond accelerating the clinical translation of nonthermal ablation procedures, the ultrafast 3D microbubble imaging techniques developed in this work stand to improve the safety and efficacy of other cavitation-mediated FUS treatments both in the brain (*e.g.*, BBB permeabilization 17-22, ultrasound-assisted tissue fractionation 72 and clot lysis 73) and in other parts of the body (*e.g.*, uterus 74, pancreas 75, heart 76). Moreover, similar methods could also be incorporated during thermoablative FUS brain therapies in which cavitation activity is unwanted 10-14 to further improve treatment safety. The use of ultrafast 3D acoustic imaging technology is therefore poised to become ubiquitous within the therapeutic ultrasound community as the field continues to develop and mature over the coming years.

## Materials and Methods

### Experimental Design

All experiments were performed with prior approval from the Animal Care Committee at Sunnybrook Research Institute (SRI) and were in accordance with the Canadian Council on Animal Care and ARRIVE (Animal Research: Reporting of *In Vivo* Experiments) guidelines. Experiments were performed on a total of seventeen New Zealand White rabbits (male, 3-4 kg; Charles River, Saint-Constance, QC, Canada) that were divided into two separate cohorts. The first cohort of animals (cohort #1: rabbits #1-9) were used to evaluate the predictive capability of ultrafast 3D microbubble imaging data to detect FUS-induced tissue damage distributions measured post-sonication. All animals in this cohort were recovered from anesthesia immediately following the FUS procedures. Seven animals (rabbits #1-7) underwent follow-up MRI at the 24 h time point, and were sacrificed either 24 h (rabbits #1) or 48 h (rabbits #2-6), post-sonication for histopathological analysis. One animal died overnight after completing the 24 h follow-up MRI scan, for which no histological data is available (rabbit #7). The remaining animals in this cohort both underwent follow-up MRI and were sacrificed 8 d (rabbits #8-9) post-sonication to examine longer-term effects from the exposures. A second cohort of animals (cohort #2: rabbits #10-17) were used to examine high volume rate intra-burst spatiotemporal microbubble dynamics *in vivo*. No MRI or histopathological data is provided for the animals in this cohort.

### Animal Preparation

Animals were housed in the SRI animal facility (Toronto, ON, Canada) with access to food and water *ad libitum*. Craniotomies (≈ 2 cm x 2 cm) were performed on the animals in cohort #1 a minimum of 2 weeks before the experiments to provide a path for the FUS beam into the brain. The skin over the craniotomy was sutured and allowed to heal completely prior to the sonications. The experiments conducted on the animals in cohort #2 were performed with intact calvaria. On the day of the FUS procedures, animals were anaesthetized via intramuscular injection of a mixture of ketamine (50 mg/kg) and xylazine (5 mg/kg) as needed to facilitate tracheal intubation, after which anesthesia was maintained with 1-3% isofluorane and medical air (2 L/min). The carrier gas used in combination with isofluorane anesthesia can have a profound impact on ultrasound-stimulated microbubble activity in the brain, and medical air has previously been shown to promote the occurrence of wideband emissions and vascular damage relative to pure oxygen 77. Hair on the animals' heads was removed with an electric razor and depilatory cream. The scalp was washed with mild soap and water to prevent chemical irritation from the cream. The ear vein was catheterized with a 22G angiocatheter. The animals were laid supine on a platform with their heads supported by a plastic membrane in contact with the degassed/deionized water-filled phased array. Degassed/deionized water provided acoustic coupling between the animal head and the membrane. Custom-built ear/bite bars stabilized and restrained the animal's head for the duration of the experiments. The animals were positioned such that the mid-thalamic region was centered on the hemispherical transducer's acoustic axis approximately 2-3 cm distal to the array's geometric focus to maximize the number of array elements with unobstructed lines of sight into the brain through the cranial window (**Figure 1A**). The animals were ventilated mechanically throughout the treatment and imaging procedures, and both heart rate and oxygen saturation levels were monitored with an MRI compatible digital pulse oximeter (8600V; Nonin Medical, Inc., Plymouth, MN, USA). Body temperature was maintained with multiple blankets and heated saline packs.

### Clinical-Scale Prototype FUS Brain System

The ultrasound exposures were delivered by an in-house designed and manufactured multi-frequency transmit/receive sparse hemispherical FUS phased array system 47. The phased array consists of 256 transducer modules distributed over a 31.8 cm diameter hemispherical aperture. Each module consists of 3 concentric cylindrical lead zirconate titanate (PZT-4) elements driven in their lateral mode at frequencies (***f***_0_) of 306, 612, and 1224 kHz (inner/outer diameter = 1.4 λ/2.0 λ, height = 1.2 λ, λ = acoustic wavelength in water), with transmit or receive functionality available for each element. The module locations were optimized to suppress grating/side lobe formation via numerical simulations 58. A custom-built 256-channel driving system was employed to excite the transmit elements, and the receiver channel data were acquired by two synchronized 128-channel data acquisition systems (SonixDAQ; Ultrasonix, Inc., Richmond, BC, Canada). Further details on the manufacturing and characterization of the FUS phased array system can be found in Ref. 47.

### FUS Treatment Protocol

FUS was applied in combination with an intravenous injection of contrast agent microbubbles using a 3D subharmonic imaging-based feedback approach 47 that was modified to enable multi-point exposure level calibration. For each animal in cohort #1, four target locations were arranged in a 2 × 2 square grid (side length = 6 mm) oriented in an axial plane centered in the mid-thalamus (**Figure 1B**). Definity^TM^ microbubbles (0.20 mL/kg, approximately 10 × dose recommended for clinical imaging) were infused through an extension line (length = 45 cm, inner diameter = 1.2 mm; Qosina, Ronkonkoma, NY, USA) connected to the ear vein catheter with an automated syringe pump (NanoJetXF Stereotaxic Syringe Pump; Chemyx, Inc., Stafford, TX, USA). The microbubble solution was infused over 90 s by pushing saline through the line beginning simultaneously with the start of each sonication. Burst-mode FUS (***f***_0_ = 612 kHz, burst length = 10 ms) was applied at each grid point via electronic beam steering of the focus (clockwise pattern viewed looking into the dome). All SPTPN pressures reported for animals in cohort #1 are estimated *in situ* based on an attenuation coefficient in brain tissue of 5 Np/m/MHz 78, an average path length in brain tissue of 1 cm, and the geometric steering loss estimated from previously reported array transmit characterization data 47, whereas additional de-rating was applied for the animals in cohort #2 (*i.e.*, no craniotomies) based on previous insertion loss measurements with *ex-vivo* rabbit calvaria (pressure transmission = 68 ± 11% at ***f***_0_ = 612 kHz 47).

The multi-point sonications (cohort #1) consisted of two stages; an initial 'calibration stage', during which the array output was varied to determine the *in-situ* pressure threshold for detecting subharmonic microbubble activity at each grid point, followed by a 'fixed-pressure stage'. During the calibration stage, burst-mode FUS was steered over the entire grid at a fixed array output and channel data were acquired at each grid point at the beginning of each burst (capture length = 3.3 ms, sampling rate = 10 MS/s) using the array elements tuned to the subharmonic of the driving frequency (*i.e.*, ***f***_0_/2 = 306 kHz). Once the entire grid was exposed and prior to increasing the array output for the next cycle of the calibration stage, the raw channel data obtained from each grid point were downloaded from the acquisition system into memory and transferred to a general-purpose GPU (ZOTAC GeForce GTX 980 Ti, 6 GB memory, 2816 cores) for processing. The receiver signals were filtered using a zero-phase digital bandpass filter (eighth order Butterworth, 200-400 kHz passband), and source field intensity distributions were reconstructed over 3D volumes centered on the target location under investigation (FOV = 10 mm x 10 mm x 20 mm, voxel size = 1.0 mm x 1.0 mm x 1.0 mm,* τ* = 3 ms) using a delay, sum, and integrate beamforming algorithm. The ultrasound transmission, data transfer, and processing times place an upper limit on the rate at which bursts can be delivered under 3D acoustic imaging-based feedback guidance. With the settings employed in cohort #1, these processes required a total of ~2.2 s for a four-point grid, resulting in a burst repetition frequency of 0.45 Hz per grid point during calibration.

Starting from an estimated SPTPN pressure of 0.20-0.25 MPa *in situ*, the applied pressure level was increased linearly at each grid point (step size ≈ 30-40 kPa) until a single distinct source (peak sidelobe ratio ≤ -3 dB 47) was observed in the 3D acoustic imaging data from one of the four targets. The pressure step size was reduced by a factor of two (≈ 15-20 kPa) following the first subharmonic detection event, and the array output at grid points for which subharmonic activity was detected (*i.e.*, triggered grid points) was set to zero for the remainder of the calibration stage. The strategy of decreasing the pressure step size following the first threshold event allows the use of a coarser step size at the outset to minimize the time until subharmonic activity is initiated *in vivo*, while mitigating the potential of overshooting the pressure applied to the remaining untriggered grid points (*i.e.*, targets at which subharmonic activity has yet to be detected during the calibration stage). The calibration stage continued until subharmonic activity was detected at all four grid points, after which each target was exposed (fixed-pressure stage: ***f***_0_ = 612 kHz, burst length = 10 ms, burst repetition frequency = 1 Hz, duration = 120 s) at a different percentage of the subharmonic peak negative pressure threshold for the corresponding location (*i.e.*, 0%, 50%, 100%, or 150% *p*_sub_). The 0% *p*_sub_ target level corresponds to the lowest possible non-zero transducer output from the FUS system (SPTPN pressure ~ 20 kPa *in-situ*). A delay of ~8 s was required between the calibration and fixed-pressure sonication stages to allow for automated re-programming of the data acquisition systems. The target locations of the different exposure levels were randomized in each animal. During the fixed-pressure stage, received channel data were captured over the entire burst length (capture length = 14 ms, sampling rate = 10 MS/s) using the elements tuned to the subharmonic frequency, and were stored for offline analysis. In each animal (cohort #1), multi-point pressure ramp sonications (step size ≈ 15-20 kPa) were performed prior to microbubble administration to gather baseline acoustic signals at each grid point.

For each animal in cohort #2, multiple successive single-point exposures were performed targeting the mid-thalamus (*n* = 22 sonications across 8 animals, 2-4 sonications per animal). The treatment parameters were otherwise identical to those employed in cohort #1, with the exception that received channel data were captured over the entire burst length during the calibration sonication stage (capture length = 13 ms, sampling rate = 10 MS/s), resulting in a burst repetition frequency of 0.5 Hz during calibration with the chosen settings (FOV = 10 mm × 10 mm × 10 mm, voxel size = 1.0 mm x 1.0 mm x 1.0 mm,* τ* = 10 ms). The sonications performed in this animal cohort were terminated automatically following subharmonic event detection (*i.e.*, no fixed-pressure sonication stage).

### Magnetic Resonance Imaging

The FUS procedures were performed under MRI guidance (see **Table 4** for sequence parameters). The platform with the animal was moved between the FUS system and a 3.0 T MRI scanner (MAGNETOM Prisma; Siemens Healthcare, Erlangen, Germany) for treatment planning and assessment of the induced tissue effects. All FUS exposures were performed outside of the MRI suite, as the prototype FUS brain system employed was not MRI-compatible. MR images were acquired (11 cm diameter loop coil) with a T_2_w sequence for target selection and detecting edema, a T_1_w sequence before and after intravenous injection of a gadolinium-based MRI contrast agent (0.1 ml/kg Gadovist^TM^; Bayer Inc., Toronto, ON, Canada) to detect changes in BBB permeability, and a T_2_*w sequence to monitor for RBC extravasations produced by the sonications 17. The MRI in-plane spatial resolution (0.4 mm x 0.4 mm) and slice thickness (1.5 mm) were both fixed across all sequence types (**Table 4**). A comprehensive set of MR images (*i.e.*, T_1_w/T_2_w/T_2_*w scans, axial and coronal planes) was acquired at the beginning and end of each treatment session, as well as during follow-up imaging. Hypointense regions within the post-sonication T_2_*w MRI volumes were segmented manually slice-by-slice for each grid point per animal (cohort #1) using the axial image series. Pre-sonication T_2_*w MRI volumes were used as a reference during segmentation to distinguish hemorrhage from anatomical structures that are hypointense in T_2_*w sequences. MRI-based segmentation was performed by three independent raters (R.M.J., D.M., and L.L.) that were unblinded to the experimental conditions, and voxels identified as hypointense in at least 2 of 3 segmentations were considered to contain hemorrhage. It was not possible to blind raters to the experimental conditions as it was obvious which regions of T_2_*w hypointensity corresponded to which target level in a given animal.

### Passive Cavitation Imaging

Source field intensity volume distributions were generated using a time-domain delay, sum, and integrate beamforming algorithm 59. With a set of received signals 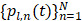 recorded during burst 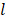 on an array of 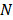 detectors with spatial positions 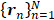, the source field intensity 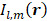 for an integration window 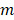 of length 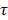 spanning 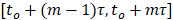 is given by:


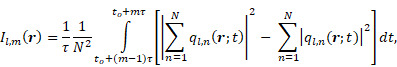


with:


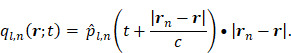


Here, 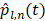 represents a temporal-filtered version of 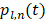, 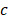 is the sound speed of the propagation medium (*i.e.*, water), 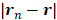 denotes the distance between receiver 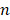 and location 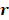 in the reconstruction grid, and 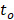 is a constant temporal offset. In this work the sound speed of water was estimated based on temperature measurements 79, and ranged from 1480-1500 m/s. The temporal origin (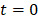) corresponds to the initiation of the ultrasound system's signal transmission and reception for a given burst. The offset term 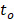 corresponds to the mean one-way travel time of sound from the array elements to the target location (*i.e.*, ~100 μs for a 15 cm distance in water).

Retrospective analysis of the acoustic emissions data acquired *in vivo* was performed offline post-sonication. In addition to conventional temporal-average processing (*i.e.*, *τ* = 3 ms/10 ms during calibration stage of cohorts #1/#2, 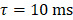 during fixed-pressure stage), short-time analysis (*i.e.*, moving, non-overlapping rectangular beamforming windows spanning FUS on-time, 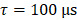) was performed to assess intra-burst microbubble dynamics during each sonication throughout both the calibration and fixed-pressure stages. In addition, microsecond-long beamforming windows (*i.e.*, 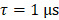) were investigated during the calibration sonication stage of a subset of animals from cohort #2. A voxel size of 0.4 mm × 0.4 mm × 0.5 mm was chosen to match the MRI in-plane spatial resolution and remain approximately isotropic. Spatial-averaging of the acoustic imaging data was performed to match the MRI slice thickness. During retrospective analysis offline, all channel data were reconstructed with the application of a zero-phase digital notch filter centered on the subharmonic frequency prior to beamforming (eighth order Butterworth, 302-310 kHz stopband), which served to isolate the broadband component of the detected acoustic emissions. Broadband emissions are a hallmark of inertial cavitation, indicating the presence of violent microbubble activity 23, and have previously been associated with tissue damage during nonthermal ablation exposures 28,32,34. Note that the notch filter was only applied during retrospective analysis, and was not incorporated intraoperatively during 3D subharmonic imaging-based exposure calibration. Lastly, all saturated receiver signals were omitted from the beamforming process for a given burst. Across all sonications carried out in animal cohort #1, 5±3 and 3±1 channels were saturated at the 150% *p*_sub_ and 100% *p*_sub_ target levels, respectively.

Ultrafast 3D PCI data reconstructed throughout multiple bursts were combined to produce sonication-aggregate 3D source intensity distributions 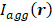
as follows:


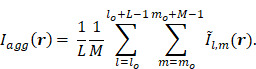


Here, 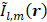 represents a spatial-filtered version of 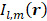 in which only the voxel corresponding to the spatial-peak source field intensity of 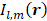, denoted by 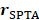, is retained as non-zero (*i.e.*, 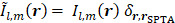, where 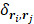 represents the Kronecker delta function), 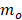 and 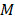 represent the first and total number of beamforming windows per burst, respectively, and 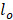 and 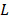 denote the first and total number of bursts per sonication, respectively. Spatial filtering provided improved performance relative to the case in which no spatial-filtering was performed (*n.b.*, equivalent to conventional whole-burst temporal averaging), potentially due to mitigation of image blur effects caused by the finite-sized point spread function of the PCI system (**Figure S4**). For the case of 100% temporal acoustic emissions sampling, *l_o_*=1, *m_o_*=1, *M*=10/*τ* (ms) and *L*=120+*L_ramp_*, with 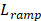 being the variable number of bursts needed during the calibration sonication stage (*i.e.*, 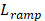
ranges from 14-22 in this study). The performance of sonication-aggregate 3D source field intensity distributions generated from temporally under-sampled acoustic emissions data were also evaluated (*i.e.*, total capture length < total FUS on-time), both with a fraction of the total burst length captured per burst (*i.e., M*<10/*τ* (ms), 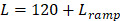) and with a subset of the total number of bursts captured per sonication (*i.e.*,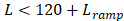, *M*=10/*τ* (ms)).

### Histopathology

At 24 h (*n* = 1, rabbit #1), 48 h (*n* = 5, rabbits #2-6), or 8 d (*n* = 2, rabbits #8-9) following sonication, the animals in cohort #1 were perfused transcardially with saline followed by 10% neutral buffered formalin. Brains were removed, post-fixed overnight at room temperature, and paraffin embedded. Axial sections (5 μm thick) were collected at 250 μm intervals and hematoxylin-eosin (H&E) stained. Sections spanning the lesion volumes, as evidenced on T_2_*w images, were imaged at 200× magnification with brightfield microscopy. Anatomical landmarks visible on both the MRI and histological data (*e.g.*, ventricular width, hippocampal shape) were used to select H&E-stained sections corresponding to regions of damage evident on T_2_*w MRI. Areas of RBC extravasations and damaged tissue, defined here as regions containing vacuolations, necrosis, glial scar formation, or hemorrhage, within the H&E-stained tissue sections were segmented manually slice-by-slice for each grid point by one researcher (D.M.) that was unblinded to the experimental settings.

### Comparison of 3D Microbubble Imaging Data with Tissue Damage Volume Distributions

The predictive capability of sonication-aggregate ultrafast 3D source field intensity distributions for detecting tissue damage volumes measured immediately post-sonication using T_2_*w MRI was assessed using ROC and PR analyses, tools for evaluating binary classifiers 71. ROC and PR analyses were performed in a voxel-wise manner in 3D using spatially co-registered PCI and MRI data. The analyses were performed in each animal (cohort #1) within a 26.8 mm × 26.8 mm × 19.5 mm FOV spanning the skull cavity (*i.e.*, spatial union of the 10.0 mm × 10.0 mm × 19.5 mm FOVs centered on each of the four grid points) on a slice-by-slice basis using the axial image series. For each slice the area of regions within the FOV consisting of true positive (TP; T_2_*w hypointense + acoustic intensity ≥ threshold), true negative (TN; not T_2_*w hypointense + acoustic intensity < threshold), false positive (FP; not T_2_*w hypointense + acoustic intensity ≥ threshold), and false negative (FN; T_2_*w hypointense + acoustic intensity < threshold) classifications were computed for a fixed source field intensity threshold, and this process was repeated for a range of acoustic intensity thresholds to generate ROC (*i.e.*, true positive rate (TPR = TP/(TP+FN)) as a function of false positive rate (FPR = FP/(FP+TN))) and PR (*i.e.*, positive predictive value (PPV = TP/(TP+FP)) as a function of TPR) curves. Note that for ROC curves, classifiers with random performance are represented by a straight diagonal line from (0,0) to (1,1), whereas for PR curves random chance classifiers are represented by a horizontal line specified by the proportion of positives in the dataset as y = P/(P+N), where P = TP+FN and N = TN+FP denote positives and negatives, respectively 71. ROC and PR curves were computed both using the entire dataset (*i.e.*, 'global') and independently for each animal in cohort #1 (*i.e.*, 'animal-wise'), and animal-wise average ROC/PR curves were generated to assess the variance in the data 80. In addition to 3D voxel-wise analysis, 2D pixel-wise classifications were performed using only the axial image data corresponding to the focal plane (see **Figure S2** for corresponding data from each animal in cohort #1). Binary classifications of tissue damage were also performed for both H&E and MRI datasets. For the binary analyses the sonication-aggregate SPTA source field intensity was computed for each target point per animal, and its predictive capability for detecting the presence of any tissue damage found throughout the associated H&E and MRI volume segmentations was evaluated. The Euclidean distance separating the centroids of the binary tissue damage volumes predicted via sonication-aggregate 3D PCI data and those measured using T_2_*w MRI was calculated for each target point for which tissue damage was evident on MRI. For the centroid calculation of the PCI data, the 'optimal' source field intensity threshold value corresponding to the maximum F_1_-score was chosen.

### Statistical Analysis

One-way analysis of variance (ANOVA) testing was applied to determine whether there were any statistically significant differences in the mean SPTPN pressure subharmonic threshold values between groups when the grid points (cohort #1) were stratified based on either the exposure level (*i.e.*, 0%, 50%, 100%, or 150% *p*_sub_) or target location within the brain (*i.e.*, anterior-left, anterior-right, posterior-left, or posterior-right grid point), followed by post-hoc Tukey multiple comparisons testing as appropriate. One-way ANOVA testing was also used to compare the mean *in-situ* subharmonic pressure threshold values for two different dosages of Definity^TM^ (0.20 ml/kg in the current study vs. 0.02 ml/kg in our previous work with a similar experimental setup, *n* = 49 of 67 values were reported in Ref. 47). The 'optimal' source field intensity thresholds were chosen from the ROC and PR curves as the values maximizing Youden's *J* statistic (*i.e.*, *J* = TPR - FPR 81) and the F_1_-score (*i.e.*, F_1_ = 2 * PPV * TPR/(PPV + TPR) 71), respectively. Area under the curve (AUC) values were computed for both ROC (AUC_ROC_) and PR (AUC_PR_) plots.

## Supplementary Material

Supplementary figures.

Supplementary movie S2.

## Figures and Tables

**Table 1:** 3D PCI data for bursts containing subharmonic activity during multi-point exposure level calibration *in vivo*

	Mean ± SD	Range [Min,Max]
*p*_sub_ (MPa)	0.67 ± 0.08	[0.55,0.87]
Intra-Grid *p*_sub_ Range (MPa)	0.14 ± 0.05	[0.06,0.29]
Detection Time (s)	31 ± 7	[20,49]
Steering Distance (mm)	27 ± 3	[21,32]
Steering Factor (%)	69 ± 3	[64,78]
Positional Error (mm)	1.1 ± 0.8	[0,3.2]
-3 dB Main Lobe Short Axis (mm)	3.1 ± 0.3	[2.5,3.6]
-3 dB Main Lobe Long Axis (mm)	7.0 ± 0.6	[5.9,8.6]

The subharmonic pressure thresholds (*p*_sub_) and intra-grid *p*_sub_ range values are *in-situ* SPTPN estimates. Steering distance denotes the distance from the target location to the array's geometric focus (*i.e.*, [0,0,0] mm in array coordinates), and the corresponding steering factor was estimated based on previously reported array transmit characterization data 47. Positional error is defined as the distance between the location of SPTA source field intensity and the intended target. The main lobe beamwidths (short/long axis sizes) are calculated based on 3D ellipsoidal fits of the -3 dB source field intensity isosurfaces. Data corresponds to a total of 36 grid points across all 9 animals in cohort #1.

**Table 2:** PCI and MRI binary tissue damage volume centroid comparison

Rabbit #	150% p_sub_ Total (mm)	150% p_sub_, Acoustic Axis (mm)	150% p_sub_, Axial Plane (mm)	100% p_sub_, Total (mm)	100% p_sub_ Acoustic Axis (mm)	100% p_sub_ Axial Plane (mm)
1	0.5	0.1	0.5	0.4	0.1	0.4
2	0.3	0.1	0.3	0.4	0.1	0.4
3	0.6	0.2	0.6	0.5	0.2	0.5
4	1.4	1.3	0.4	1.9	1.7	0.8
5	0.2	0.1	0.2	0.4	0.1	0.4
6	1.7	1.6	0.4	1.5	1.4	0.5
7	1.4	1.3	0.2	1.0	0.2	1.0
8	0.4	0.2	0.4	NA	NA	NA
9	1.7	1.6	0.4	NA	NA	NA

Euclidian distance between the centroids of the tissue damage volumes predicted via sonication-aggregate 3D PCI and those measured using T2*w MRI. Total distances are provided along with the components along the transducer's acoustic axis (superior-inferior direction) and within the orthogonal axial plane. Data are provided on an animal-wise basis (cohort #1) and are stratified based on the exposure level.

**Table 3:** Dataset imbalance for binary classification analysis

Rabbit #	3D:P(cm^3^)	3D:N(cm^3^)	3D:P/(P+N)	2D:P(cm^2^)	2D:N(cm^2^)	2D:P/(P+N)
1	0.10	14.48	0.7%	0.14	7.15	1.9%
2	0.10	14.48	0.7%	0.16	7.13	2.2%
3	0.09	14.49	0.6%	0.11	7.18	1.5%
4	0.07	14.51	0.5%	0.13	7.16	1.8%
5	0.06	14.52	0.4%	0.12	7.17	1.7%
6	0.05	14.53	0.3%	0.13	7.16	1.8%
7	0.06	14.52	0.4%	0.10	7.19	1.4%
8	0.03	14.55	0.2%	0.06	7.23	0.8%
9	0.03	14.55	0.2%	0.06	7.23	0.8%
Global	0.59	130.63	0.5%	1.01	64.60	1.6%

Class distribution for the MRI_2D_ and MRI_3D_ datasets. The positive (P = damaged tissue) and negative (N = undamaged tissue) class sizes (MRI_3D_: volume, MRI_2D_: area) are provided both on an animal-wise basis (cohort #1) and for the global datasets.

**Table 4:** MRI parameters

	T_1_w	T_2_w	T_2_*w
Sequence Type	turbo spin echo	turbo spin echo	3D gradient echo
Echo Time (ms)	8.6	82	15
Repetition Time (ms)	500	3100	27
Echo Train Length	4	8	1
Number of Averages	3	2	2
FOV (cm)	10 × 10	10 × 10	10 × 10 × 2.4
Matrix Size	256 × 256	256 × 256	256 × 256 × 16
Slice Thickness (mm)	1.5	1.5	1.5
Bandwidth (kHz)	±62.7	±15.4	±33.3
Flip Angle (°)	150	150	13

T_1_w, T_2_w, and T_2_*w MRI sequence types and parameters. All MRI data were acquired on a 3T MRI scanner (MAGNETOM Prisma 3T; Siemens Healthcare, Erlangen, Germany) using an 11 cm loop coil.

## Abbreviations

1D: one-dimensional
2D: two-dimensional
3D: three-dimensional
ANOVA: analysis of variance
AUC: area under the curve
BBB: blood-brain barrier
CNS: central nervous system
F_1_: F_1_-score
FN: false negative
FP: false positive
FPR: false positive rate
FOV: field-of-view
FUS: focused ultrasound
GPU: graphics processing unit
H&E: hematoxylin-eosin
τ: integration window length
PZT-4: lead zirconate titanate
MRI: magnetic resonance imaging
MIP: maximum intensity projection
N: negative
PCI: passive cavitation imaging
P: positive
PPV: positive predictive value
PR: precision recall
ROC: receiver operating characteristic
RBC: red blood cell
SPTA: spatial-peak temporal-average
SPTPN: spatial-peak temporal-peak negative
SD: standard deviation
*p*_sub_: subharmonic pressure threshold
SRI: Sunnybrook Research Institute
T_1_w: T_1_-weighted
T_2_w: T_2_-weighted
T_2_*w: T_2_*-weighted
*f*_0_: transmit frequency
TP: true positive
TPR: true positive rate
TN: true negative
λ: wavelength
J: Youden's index
