# Supplementary material for: Ultrafast three-dimensional microbubble imaging in vivo predicts tissue damage volume distributions during nonthermal brain ablation
Source: Theranostics. 2020 Jun 1;10(16):7211–30. doi: 10.7150/thno.47281 (PMC7330857; doi:10.7150/thno.47281)
Supplement: Supplementary file 1 — Supplementary figures. [file thnov10p7211s1.pdf]

Ultrafast three-dimensional microbubble imaging *in vivo* predicts tissue damage volume distributions during nonthermal brain ablation

Ryan M. Jones[1], Dallan McMahon[1,2], and Kullervo Hynynen[1,2,3]

[1]*Physical Sciences Platform, Sunnybrook Research Institute, Toronto, Ontario, Canada*

[2]*Department of Medical Biophysics, University of Toronto, Toronto, Ontario, Canada*

[3]*Institute of Biomaterials and Biomedical Engineering, University of Toronto, Toronto, Ontario, Canada*

Supplementary Material

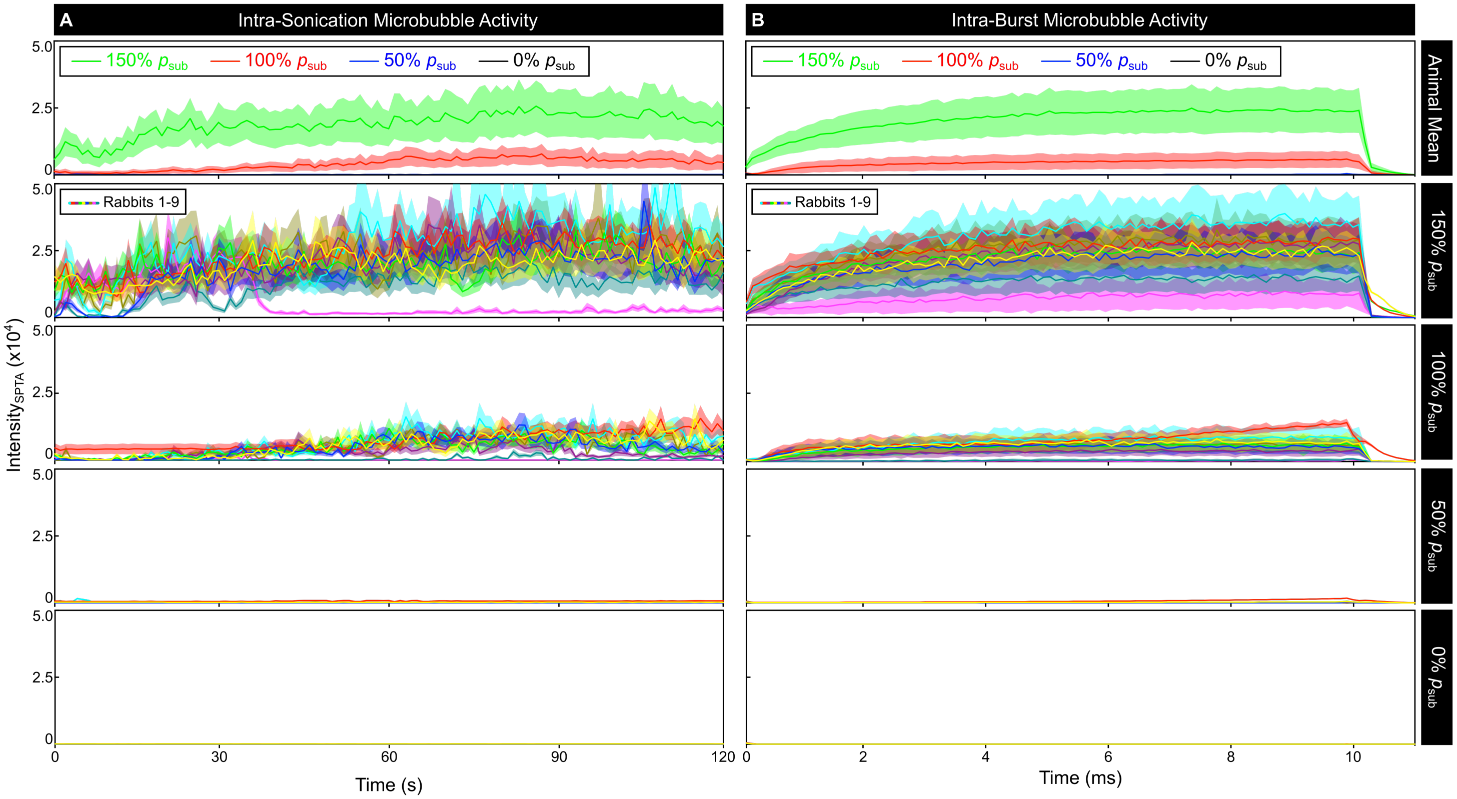

Figure S1. Animal-wise intra-sonication and intra-burst microbubble activity. (**A**) SPTA source field intensity as a function of time throughout the 120 s fixed-pressure sonication stage for each target level (0%, 50%, 100%, and 150% p_{sub}) of each animal in cohort #1, along with an animal-wise mean per target level. The shaded regions denote ± 0.5 SDs from the mean, calculated throughout each 10 ms burst length (τ = 100 µs). (**B**) SPTA source field intensity as a function of time throughout the 10 ms burst length (τ = 100 µs) for each target level (0%, 50%, 100%, and 150% p_{sub}) of each animal in cohort #1, along with an animal-wise mean per target level. The shaded regions denote ± 0.5 SDs from the mean, calculated throughout each 120 s exposure. In (A,B), error bars of ± 0.5 SDs from the mean were chosen for data visualization purposes.

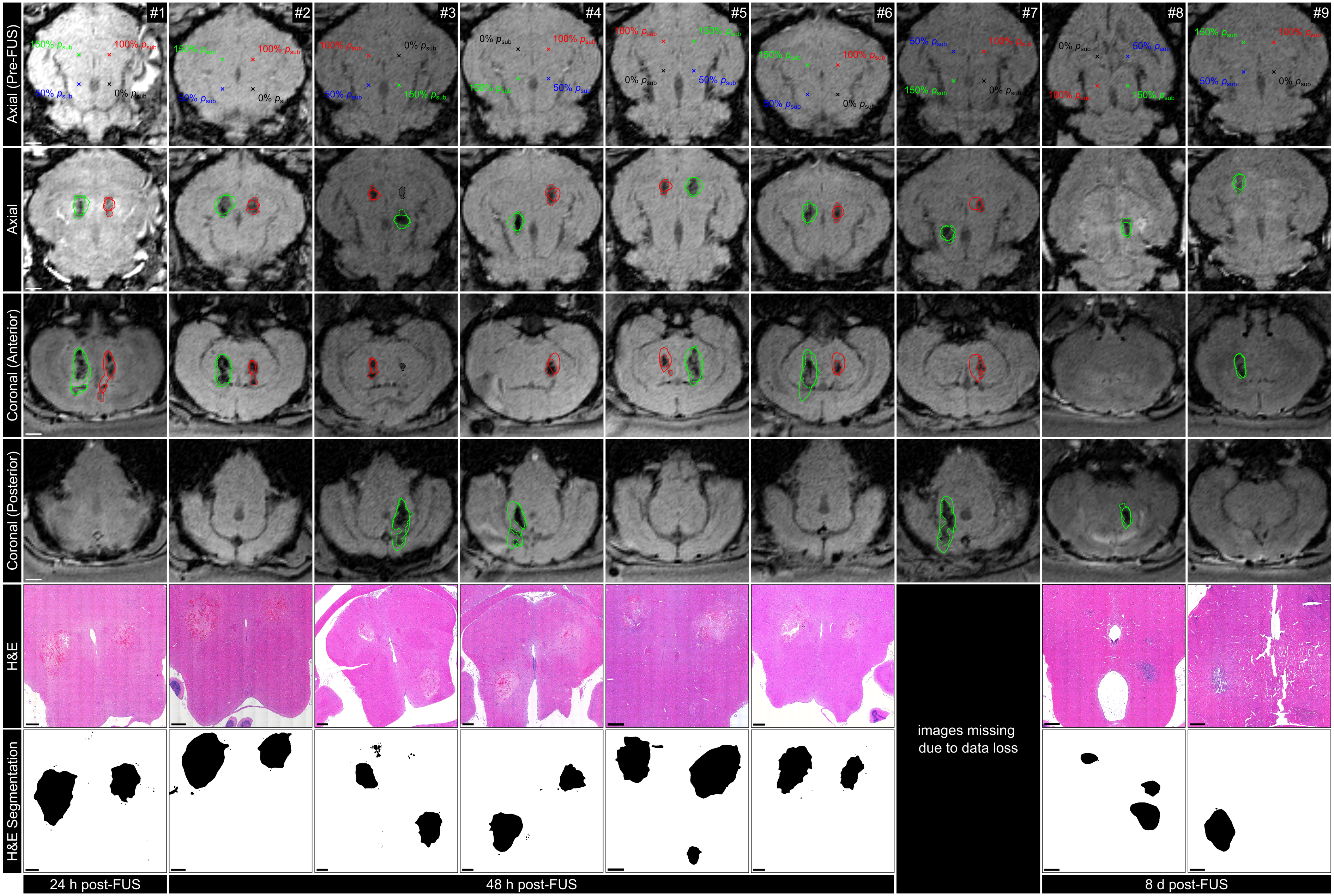

Axial (Pre-FUS)
Axial
Coronal (Anterior)
Coronal (Posterior)
H&E
H&E Segmentation
#1
#2
#3
#4
#5
#6
#7
#8
#9
150% p_{sub}
100% p_{sub}
50% p_{sub}
0% p_{sub}
images missing
due to data loss
24 h post-FUS
48 h post-FUS
8 d post-FUS

Figure S2. Animal-wise MRI, PCI, and H&E data. T_2*w MRI data (axial and coronal planes) acquired pre- and immediately post-sonication for each animal in cohort #1 (rabbits #1-9). Color-coded target locations and their corresponding target levels are overlaid on the pre-sonication axial T_2*w scans (green = 150% p_{sub}, red = 100% p_{sub}, blue = 50% p_{sub}, black = 0% p_{sub}). Segmented regions of T_2*w MRI signal hypointensity induced by the exposures (dashed lines) and corresponding sonication-aggregate ultrafast PCI contour data at the operating threshold that maximizes the F_1-score of the global PR curve of the MRI_{3D} dataset (solid lines) are overlaid and are both color-coded by target level. Axial H&E stained tissue sections and binary segmented regions of damage are provided for the planes corresponding to the axial MRI data. The sacrifice time points associated with the H&E data are provided for each rabbit. Scale bars on the MRI (H&E) data indicate 5 mm (1 mm).

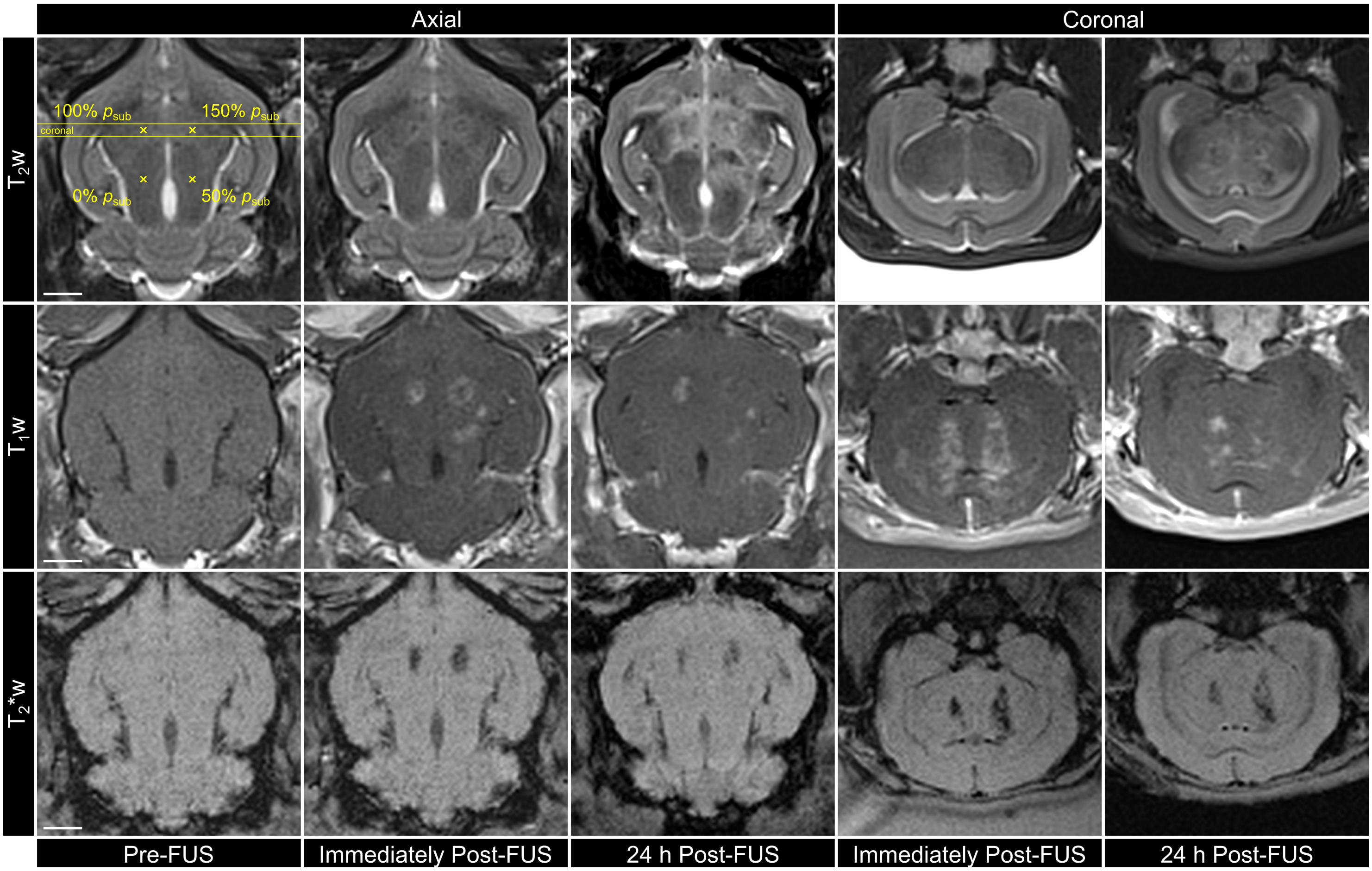

Figure S3. Multi-sequence MRI-based assessment of acute treatment response during microbubble-mediated nonthermal brain ablation. MRI data (axial and coronal planes) from three different sequence types (T_2*w, T_1w, and T_2w) acquired pre-sonication, immediately post-sonication, and 24 h post-sonication of rabbit #5. Post-sonication T_1w scans were acquired immediately following administration of a gadolinium-based MRI contrast agent (0.1 ml/kg Gadovist™). Target locations, their corresponding target levels, and the coronal slice volume are overlaid on the pre-sonication axial T_2w scan in yellow. Scale bars indicate 5 mm.

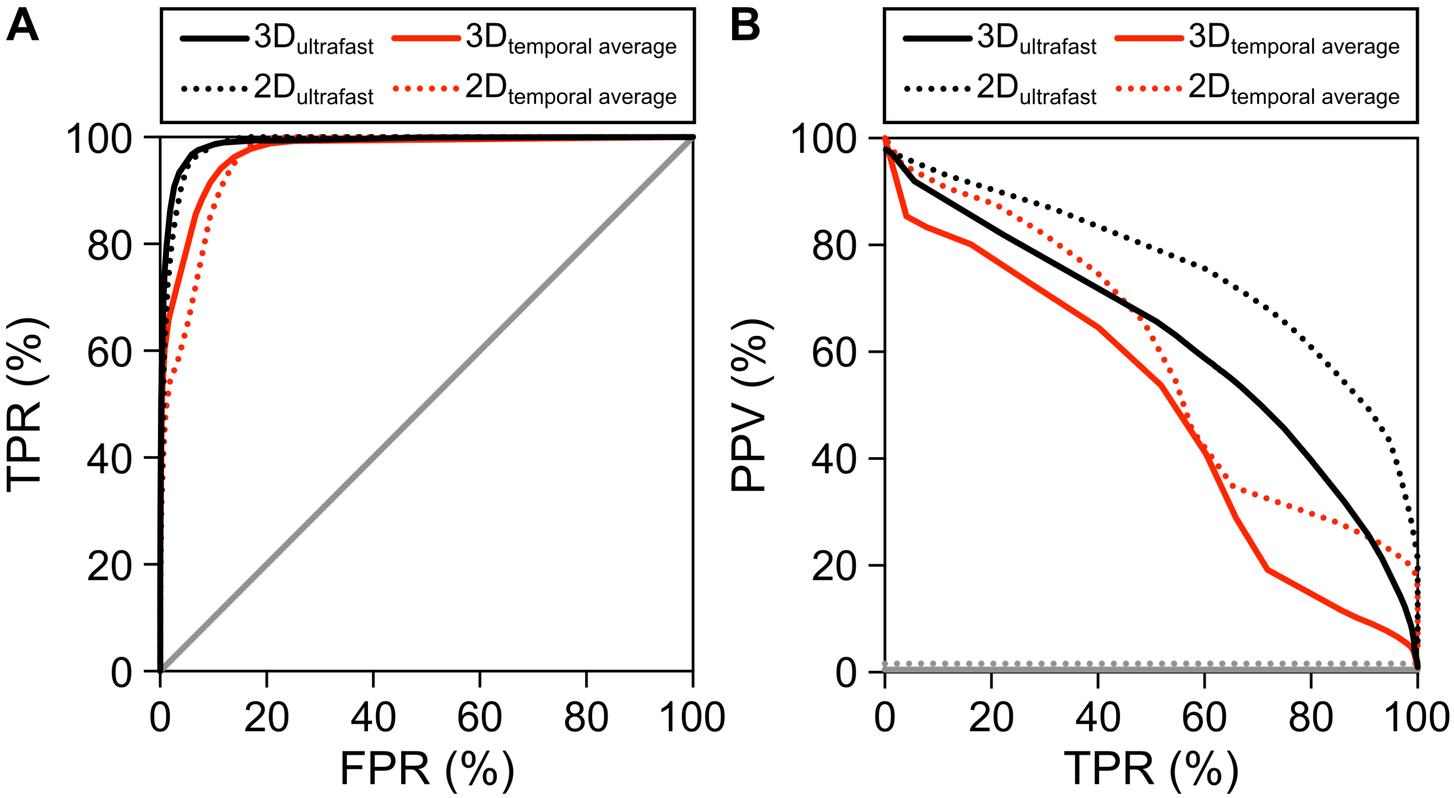

Figure S4. Comparison of ultrafast and temporal-average processing methods for predicting FUS-induced brain tissue damage distributions. Global (**A**) ROC and (**B**) PR curves for MRI-based classification of tissue damage (MRI_{2D} and MRI_{3D} datasets) via sonication-aggregate 3D PCI data generated using both ultrafast processing (10 kHz imaging volume rate) and conventional whole-burst temporal averaging. (A,B) Gray lines denote the ROC/PR curves for classifiers with random performance for a specific dataset (solid = 3D, dotted = 2D). TPR = True Positive Rate, FPR = False Positive Rate, PPV = Positive Predictive Value.

Movie S1. Megahertz-rate 3D microbubble imaging in vivo. (Left Column) Axial (XY-plane) and coronal (XZ-plane) views of -3 dB source field intensity isosurfaces at a 1 MHz volume rate (τ = 1 μs) over the course of the ultrasound burst of the calibration sonication during which subharmonic microbubble activity was first detected intraoperatively (rabbit #11, in-situ SPTPN pressure ~0.66 MPa). 3D PCI data were computed for every 10th frame (10 μs separation between frames) and displayed only during integration windows with a peak sidelobe ratio ≤ -3 dB. (Right Column) Whole-burst temporal-average -3 dB source field intensity isosurface (τ = 10 ms) corresponding to the same ultrasound burst.
